# Homeostasis of mRNA concentrations through coupling transcription, export, and degradation

**DOI:** 10.1016/j.isci.2024.110531

**Published:** 2024-07-18

**Authors:** Qirun Wang, Jie Lin

**Affiliations:** 1 Center for Quantitative Biology, Academy for Advanced Interdisciplinary Studies, Peking University, Beijing 100871, China; 2 Peking-Tsinghua Center for Life Sciences, Academy for Advanced Interdisciplinary Studies, Peking University, Beijing 100871, China

**Keywords:** Biological sciences, Neuroscience, Sensory neuroscience, Cognitive neuroscience

## Abstract

Many experiments showed that eukaryotic cells maintain a constant mRNA concentration upon various perturbations by actively regulating mRNA production and degradation rates, known as mRNA buffering. However, the underlying mechanism is still unknown. In this work, we unveil a mechanistic model of mRNA buffering: the releasing-shuttling (RS) model. The model incorporates two crucial proteins, X and Y, which play several roles, including transcription, decay, and export factors, in the different stages of mRNA metabolism. The RS model predicts the constant mRNA concentration under genome-wide genetic perturbations and cell volume changes, the slowed-down mRNA degradation after Pol II depletion, and the temporal transcription dynamics after exonuclease depletion, in agreement with multiple experiments. Finally, we present a list of X and Y candidates and propose an experimental method to identify X. Our work uncovers potentially universal pathways coupling transcription, export, and degradation that help cells maintain mRNA homeostasis.

## Highlights

• The releasing-shuttling model unifies multiple experimental observations
• Multi-functional proteins synchronize mRNA production, export, and degradation rate

## Graphical abstract

**Figure:**
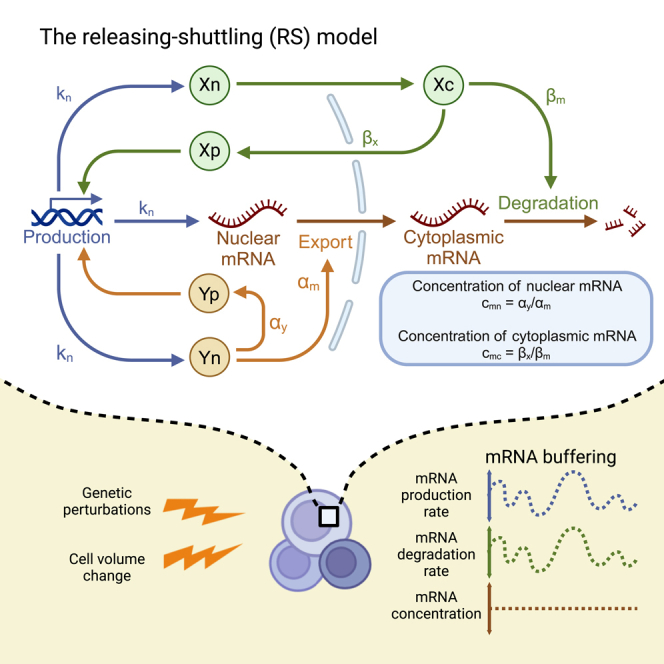

## Introduction

Maintaining an appropriate mRNA level is vital for all living cells. Surprisingly, many eukaryotic cells adjust the mRNA production rate and the mRNA degradation rate to achieve a constant mRNA concentration, an elegant but mysterious phenomenon called mRNA buffering.^1^^,^^2^^,^^3^ Multiple experiments carried out in the budding yeast *Saccharomyces cerevisiae* showed that the total mRNA concentration and also the mRNA concentrations of most individual genes are invariant against various genetic perturbations.^4^^,^^5^^,^^6^^,^^7^^,^^8^^,^^9^^,^^10^^,^^11^^,^^12^^,^^13^ In cases where transcription-related genes are perturbed, the mRNA production rate decreases; interestingly, the mRNA degradation rate decreases accordingly so that the mRNA concentration is invariant. In cases where mRNA degradation-related genes are perturbed, the mRNA degradation rate drops, and the production rate decreases accordingly to maintain a constant mRNA concentration. Beyond the steady-state results, Chappleboim et al. recently showed that, after an acute depletion of mRNA degradation factors, the mRNA degradation rate dropped immediately. However, the production rate decreased after a delay, so the total mRNA concentration temporarily accumulated and then gradually returned to its original level.^14^ mRNA buffering phenomenon has been reported in mammalian cells as well,^15^^,^^16^ suggesting its universality across eukaryotes. Strikingly, Berry et al. subjected mammalian cells to genome-wide genetic perturbation screening and found that the total mRNA concentration in both the nucleus and cytoplasm remained virtually unchanged despite a significant variation in the mRNA production rate.^17^

Meanwhile, the total mRNA concentration and the mRNA concentrations of most genes are also constant as the cell volume increases.^17^^,^^18^^,^^19^^,^^20^ The homeostasis of mRNA concentration in a growing cell volume is often considered a consequence of an mRNA production rate proportional to cell volume with a constant mRNA degradation rate.^21^^,^^22^ The volume-scaling mRNA production rate has been proposed to come from the limiting nature of polymerase II (Pol II)^23^^,^^24^—the mRNA production rate is proportional to the copy number of RNA Pol II, which is proportional to the cell volume. However, Mena et al. found that the mRNA production rate is constant as the cell volume increases in *Saccharomyces cerevisiae*; nevertheless, homeostasis of mRNA concentration is still maintained due to the decreased mRNA degradation rate.^25^ Similarly, Swaffer et al. found that the mRNA production rate increases sublinearly with the cell volume in *Saccharomyces cerevisiae*. In the meantime, the mRNA degradation rate decreases accordingly and balances the mRNA production rate so that the mRNA concentration remains nearly constant.^26^

In all the aforementioned examples, a coupling between mRNA production and mRNA degradation exists and balances the two rates so that the mRNA concentration is constant. It has been suggested that mRNA feedback to its own production or degradation may lead to mRNA buffering.^14^^,^^17^^,^^26^ In addition to mRNA itself, several proteins have been suggested as possible players in mRNA buffering. One notable protein is Xrn1, the 5′-3′ exonuclease that plays a crucial role in cytoplasmic mRNA degradation. Recent studies have shown that it also shuttles into the nucleus and functions in transcription regulation, suggesting its potential role in connecting mRNA production and degradation.^8^^,^^9^^,^^27^^,^^28^^,^^29^ Similarly, the Ccr4-Not complex involved in mRNA deadenylation has also been found to shuttle between cytoplasm and nucleus and regulate transcription.^30^ Other examples include Snf1, the yeast ortholog of AMP-activated protein kinase (AMPK), which interacts with both degradation machinery and transcription factors,^1^ and Rpb4/7, which are subunits of Pol II and participate in both transcription and degradation of certain mRNAs.^4^^,^^31^^,^^32^^,^^33^ All these factors share two common features: they play a role in both transcription and mRNA degradation and can shuttle between nucleus and cytoplasm to transmit information along the mRNA metabolic pipeline. These features are necessary to connect transcription and degradation in mRNA buffering.

Despite extensive knowledge of molecular details that may contribute to mRNA buffering, its mechanism remains mysterious. In this work, we provide a new mechanistic model of mRNA buffering that can explain and unify multiple experimental observations of mRNA buffering. In this work, we mainly study the total concentration of all mRNAs, which we call the mRNA concentration in the following unless otherwise mentioned. Our conclusions regarding mRNA buffering do not necessarily apply to all individual genes since they can be under specific regulations.^8^^,^^9^^,^^17^^,^^34^^,^^35^ Although most genes exhibit constant mRNA and protein concentration as the cell volume increases, we have shown before that genes with strong (weak) promoters may exhibit sublinear (superlinear) volume scaling of mRNA and protein concentrations.^24^ In the following, we show that models involving mRNA feedback to its production or degradation cannot achieve robust mRNA buffering. We then introduce the simple model of mRNA buffering, the releasing-shuttling (RS) model. The critical ingredients of the RS model are two proteins, X and Y, released after each transcription initiation. Protein X, responsible for mRNA degradation, is exported to the cytoplasm and shuttles back to the nucleus. Protein Y is responsible for the export of mRNA to the cytoplasm. We demonstrate how the RS model quantitatively explains multiple experimental observations and makes experimentally testable predictions. Finally, we identify candidates for the essential proteins X and Y and discuss possible extensions of the RS model.

## Results

### The RS model

It has been suggested that a negative feedback of mRNA to its own production or a positive feedback to its own degradation can be potential mechanisms of mRNA buffering.^14^^,^^17^^,^^26^ However, we found that the feedback models generally cannot lead to robust buffering: a finite correlation exists between the mRNA production and the mRNA concentration (STAR methods and Figure S1). In contrast, the mRNA production rate and the mRNA concentration appeared uncorrelated across genome-wide perturbations in mammalian cells.^17^

**Figure 1:**
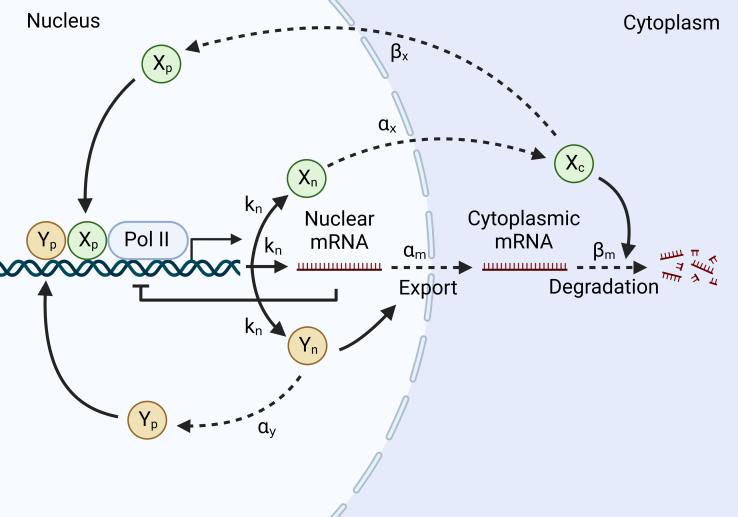
Schematic of the releasing-shuttling model One copy of protein X and Y in the released states (Xn and Yn) is released after each transcription initiation. $k_{n}$ is the mRNA production rate, which is also the rate of transcription initiation. Xn transitions to the cytoplasm with a constant rate $\alpha_{x}$ and becomes Xc in the DF state, which degrades mRNAs with a rate proportional to the constant $\beta_{m}$. Xc has a constant rate $\beta_{x}$ to shuttle back to the nucleus and becomes Xp in the TF state. Yn exports nuclear mRNAs to the cytoplasm with a rate proportional to the constant $\alpha_{m}$. Yn transitions to Yp in the TF state with a constant rate $\alpha_{y}$.

In the following, we introduce a simple model of mRNA buffering with two essential proteins, X and Y (Figure 1). X is a degradation factor that shuttles between the nucleus and the cytoplasm. Y is an export factor and is localized in the nucleus. X can be in three states: the transcription-factor (TF) state in the nucleus that is part of the preinitiation complex (PIC), Xp; the released state separated from the PIC right after transcription initiation and ready to be exported to the cytoplasm, Xn; and the decay-factor (DF) state in the cytoplasm responsible for mRNA degradation, Xc. Y can be in two states: the TF state in the nucleus that is part of the PIC, Yp, and the export-factor (EF) state released from the PIC right after transcription initiation and ready to export nuclear mRNAs to the cytoplasm, Yn. We assume that the DF state Xc has a finite rate to shuttle back to the nucleus and become the TF state Xp, supported by multiple pieces of evidence showing that mRNA decay factors shuttle in and out of the nucleus and appear critical for transcription.^1^^,^^8^^,^^9^^,^^30^^,^^31^^,^^36^ Similarly, we assume the EF state Yn has a finite rate to transition back to the TF state Yp.

The release of proteins X and Y from the PIC is justified by the fact that transcription initiation is a step-by-step process propelled by different functional groups, which sequentially bind and leave the transcription machinery. In particular, the carboxyl-terminal domain (CTD) region of Pol II is a versatile harbor providing numerous docking points for proteins functioning in diverse processes such as initiation-to-elongation transformation and mRNA processing.^37^ We suppose proteins X and Y are two of the signaling molecules that are released during transcription initiation. Recent studies show that mRNA export is under sophisticated regulations.^38^^,^^39^ Interestingly, transcription is necessary for mRNA export,^40^ supporting our model assumption. We hypothesize that protein Y in the state Yn is an export regulator of mRNA.

The mathematical equations of the RS model are the following:

(Equation 1) $$\frac{dN_{mn}}{dt}=k_{n}-\alpha_{m}\frac{N_{Yn}}{V_{n}}N_{mn}\text{,}$$

(Equation 2) $$\frac{dN_{Xn}}{dt}=k_{n}-\alpha_{x}N_{Xn}\text{,}$$

(Equation 3) $$\frac{dN_{Yn}}{dt}=k_{n}-\alpha_{y}N_{Yn}\text{,}$$

(Equation 4) $$\frac{dN_{mc}}{dt}=\alpha_{m}\frac{N_{Yn}}{V_{n}}N_{mn}-\beta_{m}\frac{N_{Xc}}{V_{c}}N_{mc}\text{,}$$

(Equation 5) $$\frac{dN_{Xc}}{dt}=\alpha_{x}N_{Xn}-\beta_{x}N_{Xc}\text{,}$$

(Equation 6) $$\frac{dN_{Xp}}{dt}=\beta_{x}N_{Xc}-k_{n}\text{,}$$

(Equation 7) $$\frac{dN_{Yp}}{dt}=\alpha_{y}N_{Yn}-k_{n}\text{.}$$

Here, $N_{Xp}$, $N_{Xn}$, $N_{Xc}$, $N_{Yp}$, $N_{Yc}$, $N_{mn}$, and $N_{mc}$ all represent copy numbers. $N_{mn}$ is the number of nuclear mRNAs, and $N_{mc}$ is the number of cytoplasmic mRNAs. $k_{n}$ is the mRNA production rate (number per unit time), which depends on multiple factors, including Pol II, Xp, and Yp (see the next section). $V_{c}$ is the cytoplasmic volume, and $V_{n}$ is the nuclear volume that is proportional to $V_{c}$^41^. The binding rate of mRNA to the EF state Yn is proportional to its concentration $N_{Yn}/V_{n}$ with a factor $\alpha_{m}$, which is the limiting step of mRNA export. $\alpha_{x}$ quantifies how fast Xn escapes the nucleus, and $\alpha_{y}$ quantifies how fast Yn transforms back to Yp. Similarly, the binding rate of mRNA to the DF state Xc is proportional to its concentration $N_{Xc}/V_{c}$ with a factor $\beta_{m}$. $\beta_{x}$ quantifies how fast Xc shuttles back to the nucleus.

In the steady states where all the time derivatives in Equations 1, 2, 3, 4, 5, 6, and 7 are zero, it is straightforward to find the solution of the RS model:

(Equation 8) $$\frac{N_{mn}}{V_{n}}=\frac{\alpha_{y}}{\alpha_{m}}\text{,}$$

(Equation 9) $$\frac{N_{mc}}{V_{c}}=\frac{\beta_{x}}{\beta_{m}}\text{,}$$

(Equation 10) $$N_{Xn}=\frac{k_{n}}{\alpha_{x}}\text{,}$$

(Equation 11) $$N_{Xc}=\frac{k_{n}}{\beta_{x}}\text{,}$$

(Equation 12) $$N_{Yn}=\frac{k_{n}}{\alpha_{y}}\text{.}$$

Intriguingly, the numbers of nuclear mRNAs $N_{mn}$ and cytoplasmic mRNAs $N_{mc}$ are proportional to the nuclear volumes $V_{n}$ and cytoplasmic volumes $V_{c}$, respectively. These are necessary conditions for any model to be biologically valid: the model must predict a constant mRNA concentration. Surprisingly, both the nuclear and cytoplasmic mRNA concentrations are independent of the mRNA production rate $k_{n}$: the RS model successfully explains the robust mRNA concentration homeostasis against the change of mRNA production rate. This is because the information on the mRNA production rate is conveyed through the amounts of Yn and Xc, synchronizing the speed of mRNA export and degradation. To ensure the number of DF state X and EF state Y to be proportional to $k_{n}$ (Equations 11 and 12), we concluded that proteins X and Y must be necessary for transcription initiation; otherwise, mRNA buffering is invalid. To explicitly demonstrate this point, we introduced a modified model in which proteins X and Y are not necessary factors to initiate transcription and found that mRNA buffering in this modified model does not hold (STAR methods and Figure S2).

Furthermore, it is conceivable that X and Y may also be released at various points throughout the transcription process as research indicates that Ccr4-Not and Xrn1 exert control over not only transcription initiation but also transcription elongation stages.^28^^,^^42^ We remark that the exact releasing timings of proteins X and Y are not critical to the predictions of the RS model as long as the production rates of Xn and Yn are proportional to the mRNA production rate. Also, the nuclear localization of protein Y is not essential for our conclusions. In a modified model in which Y is exported out of the nucleus and then shuttles back, mRNA buffering is still valid (STAR methods and Figure S3). The export and shuttling of protein X may depend on some transport factors beyond our model. Further investigation into the molecular partners facilitating these processes will provide valuable insights into the regulatory mechanisms governing mRNA homeostasis.

### The RS model predicts mRNA buffering against various genetic perturbations

The RS model shows that the validity of mRNA buffering does not require a particular form of the mRNA production rate $k_{n}$ since it does not enter the expressions of $N_{mn}$ and $N_{mc}$. Nevertheless, starting with a biologically valid model for transcription makes it possible to compare theories with experiments explicitly, as we show in the following. Therefore, we incorporated several general features of transcription regulation into the RS model. First, the copy number of Pol II is often limiting for transcription, supported by multiple experiments.^20^^,^^21^^,^^26^ Because the Pol II copy number is proportional to the nuclear volume,^26^ we used the nuclear volume as a proxy for Pol II. It has been found that the number of actively transcribing Pol II increases sublinearly with the total Pol II number given fixed gene copy numbers,^26^ presumably due to the finite DNA substrates for Pol II to bind.^24^ Therefore, we modeled the effects of limiting Pol II resource through an mRNA production rate that is a Michaelis-Menten (MM) function of the nuclear volume, which saturates for a large nuclear volume given a fixed amount of DNA. Second, the nuclear mRNAs have negative feedback to their own production,^17^^,^^43^ although the detailed mechanisms are still unclear. To sum up, we proposed the following form of the mRNA production rate:

(Equation 13) $$k_{n}=k_{0}\frac{V_{n}}{V_{n}+K_{v}}\frac{K_{m}}{c_{mn}+K_{m}}\frac{c_{Xp}}{c_{Xp}+K_{x}}\frac{c_{Yp}}{c_{Yp}+K_{y}}\text{.}$$

Here, the prefactor $k_{0}$ is a constant, and the first term on the right side of Equation 13 represents the sublinear volume dependence of actively transcribing Pol II. The second term represents the negative feedback of nuclear mRNA to transcription where $c_{mn}=N_{mn}/V_{n}$. Finally, the last two terms represent the binding probabilities of the transcription factors Xp and Yp to the PIC. Here, $c_{Xp}$ and $c_{Yp}$ are the nuclear concentrations of Xp and Yp, respectively: $c_{Xp}=N_{Xp}/V_{n}$ and $c_{Yp}=N_{Yp}/V_{n}$. In the steady state, given the total copy number of protein X ($N_{Xt}$) and the total copy number of protein Y ($N_{Yt}$), $N_{Xn}$, $N_{Xp}$, $N_{Xc}$, $N_{Yn}$, and $N_{Yp}$ can be found through the conservation equations, $N_{Xt}=N_{Xn}+N_{Xc}+N_{Xp}$, $N_{Yt}=N_{Yn}+N_{Yp}$. Here, $N_{Xn}=k_{n}/\alpha_{x}$, $N_{Xc}=k_{n}/\beta_{x}$, $N_{Yn}=k_{n}/\alpha_{y}$, and $k_{n}$ is a function of $N_{Xp}$ and $N_{Yp}$ via Equation 13.

**Figure 2:**
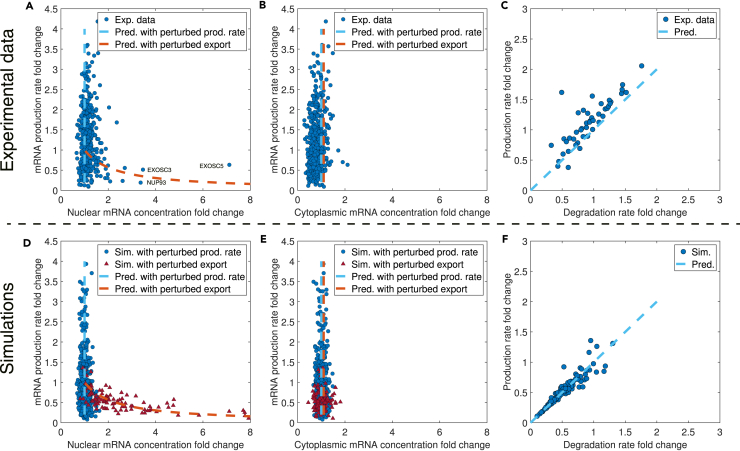
The RS model predicts mRNA buffering against genetic perturbations Exp., experiment; pred., prediction; prod., production; sim., simulation. (A) Experimental data from Berry et al.^17^ show that the nuclear mRNA concentration is buffered against changes in the mRNA production rate. Each data point represents a knockdown of a particular gene with the three genes EXOSC3, EXOSC5, and NUP93 highlighted. The blue dashed line is an x=1 line, and the red dashed line represents the prediction when nuclear mRNA export is impaired, corresponding to perturbing $\alpha_{m}$ in the RS model. The fold change represents the relative mRNA level of the cells with a gene knocked out compared to that in the control groups of cells. The same meanings of dashed lines apply to (B, D, and E). (B) Experimental data from Berry et al.^17^ show that the cytoplasmic mRNA concentration is buffered against changes in the mRNA production rate. (C) Experimental data of the mRNA production rate vs. the degradation rate from Sun et al.^9^ The blue dashed line is a y=x line. (D) Simulations of the RS model show that the nuclear mRNA concentration is buffered against changes in the mRNA production rate. The blue circles represent simulation results with multiple parameters perturbed, and the red triangles represent simulation results with $\alpha_{m}$ perturbed. The fold change represents the relative rate of the cells with genetic perturbations compared to that in the control groups of cells. The same meanings of points apply to (E). Here, we randomly sampled the mRNA copy numbers from a Poisson distribution with the means equal to the predictions of the RS model, mimicking gene expression noise. We also added Gaussian noise on top of the mRNA production rates so that the coefficients of variation (CVs) equal 0.05. The same noises were applied to (E). (E) Numerical data of the RS model show that the cytoplasmic mRNA concentration is buffered against changes in the mRNA production rate and perturbations in the mRNA nuclear export pathway. (F) Numerical data of the mRNA production rate vs. the degradation rate from the RS model. The blue dashed line is a y=x line. Data are from the same simulations of (D and E). For both the production and degradation rates, we added Gaussian noises on top of them so that the CVs equal 0.05.

To mimic genome-wide genetic perturbations similar to previous experiments,^8^^,^^9^^,^^17^ we systematically perturbed the parameters in Equation 13 of the RS model, as well as the total copy numbers of proteins X and Y (see details of numerical calculations in STAR methods). According to the RS model, all the perturbations may change the mRNA production rate but should leave the nuclear and cytoplasmic mRNA concentrations invariant. Indeed, we found that the simulated data nicely matched the experimental data (Figures 2A, 2B, 2D, and 2E),^17^ both exhibiting a virtually zero correlation between the mRNA production rate and the mRNA concentrations. In Figures 2A, 2B, 2D, and 2E, the blue dashed lines are x=1 lines, while the red dashed lines are from solving the RS model numerically with different $\alpha_{m}$, which we explain in the next section. Sun et al.^9^ measured the mRNA production rate and degradation rate and found an approximately linear relation between these two rates across 46 yeast deletion strains, which suggested mRNA buffering (Figure 2C). To further verify the RS model, we plotted the mRNA production rate vs. the mRNA degradation rate from the same simulations as Figures 2D and 2E and also found a linear relationship between them (Figure 2F) in concert with experiments.

In this work, we used a set of basic parameters (Table 1) for all simulations unless otherwise mentioned (STAR methods). The basic parameters were from the fitting of kinetic data of budding yeast with the MM constant $K_{v}$ inferred from the experimental data of mRNA production rate vs. cell volume, which we explain in later sections. These parameters are presumably different from mammalian cells. Nevertheless, using the set of basic parameters and only adjusting the $k_{0}$ value in Equation 13, we found that the simulations and experimental data already quantitatively agreed (Figure 2). Therefore, our conclusions regarding the robustness of mRNA buffering are insensitive to the parameters.

**Table 1:** The basic set of parameters

Parameter	Value	Unit
$k_{0}$	6000.00	min^−1^
$K_{x}$	180.18	fL^−1^
$K_{y}$	100.00	fL^−1^
$K_{m}$	5086.24	fL^−1^
$K_{v}$	5.83	fL
$\alpha_{m}$	3.28 × 10^−5^	fL · min^−1^
$\alpha_{x}$	0.10	min^−1^
$\alpha_{y}$	0.52	min^−1^
$\beta_{m}$	9.92 × 10^−6^	fL · min^−1^
$\beta_{x}$	2.24 × 10^−3^	min^−1^
$N_{Xt}$	2.30 × 10^5^	–
$N_{Yt}$	1.10 × 10^4^	–
$V_{n}$	2.80	fL
$V_{c}$	37.20	fL

**Figure 3:**
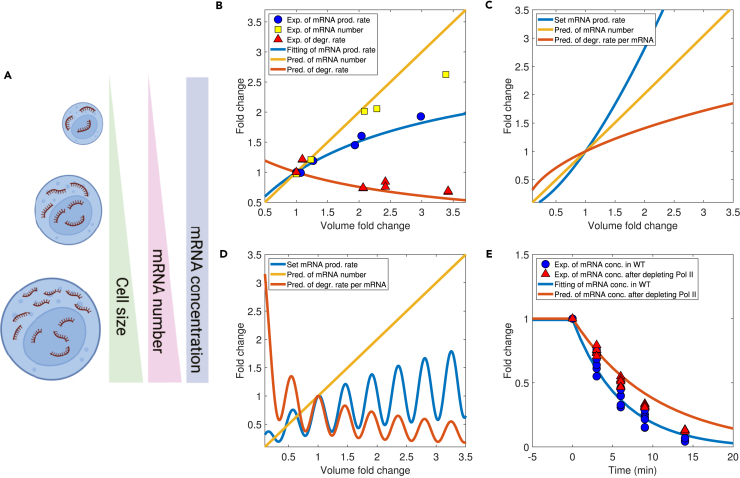
The RS model predicts mRNA buffering during cell growth and slowed mRNA degradation after Pol II depletion Exp., experiment; degr., degradation; pred., prediction; conc., concentration. (A) Schematic showing the homeostasis of mRNA concentration during cell growth. (B) Experimental data from ref. 26 for the mRNA production rate, degradation rate, and the mRNA number as a function of cell volume during cell growth. The blue line is a fitting of the experimental data ($r^{2}=0.98$). The red line is the predicted mRNA degradation rate per mRNA without any fitting parameters from the RS model. The yellow line is the predicted mRNA copy number. The fold changes represent the relative quantities (i.e., rates, molecular numbers, and volumes) of the size mutants compared to those in WT cells. The same meanings apply to (C) and (D). (C) mRNA buffering occurs even if the mRNA production rate exhibits a superlinear scaling with cell volume. In this case, the mRNA degradation rate increases with the cell volume accordingly to make the mRNA concentration constant. (D) mRNA buffering occurs even if the mRNA production rate oscillates with cell volume. In this case, the mRNA degradation rate also oscillates, and the mRNA concentration remains constant. (E) The normalized mRNA concentration for a particular gene following transcription shut-off. The blue circles are for cells without Pol II depletion, and the red triangles are for cells with half Pol II depleted. The experimental data are from Swaffer et al.^26^ The blue line is a fitting of the experimental data by the RS model ($E^{2}=3.87\times 10^{-11}$). The red line is the prediction from the RS model for cells with half Pol II depleted without further fitting. The fold changes represent the relative concentrations of mRNA compared to those before Pol II depletion.

### The RS model predicts when mRNA buffering breaks down

Experimentally, violations of nuclear mRNA buffering were observed when the export of nuclear mRNA was impaired.^17^ Intriguingly, the RS model also predicts the breakdown of nuclear mRNA buffering. Specifically, the RS model predicts that, when nuclear mRNA export is impaired, that is, a lower $\alpha_{m}$ in the RS model (Equation 8), the nuclear mRNA concentration is not constant anymore but negatively correlates with the mRNA production rate. In Figures 2A and 2D, the red dashed lines were from the numerical calculations of the RS model with changing $\alpha_{m}$. The red triangles in Figure 2D were obtained by randomly sampling $\alpha_{m}$ (see details of numerical calculations in STAR methods). In the RS model, the negative correlation between the mRNA production rate and the nuclear mRNA concentration comes from the negative feedback from the nuclear mRNA concentration to the mRNA production rate, and we confirmed this by showing that the negative correlation is absent in a modified model without this negative feedback (Figure S4). Intriguingly, the RS model predicts that the cytoplasmic mRNA concentration is uninfluenced by the impairment of nuclear mRNA export (Equation 9), which exactly matches the experimental observations (Figures 2B and 2E).

Biologically, nuclear mRNA export needs accurate mRNA processing and the proper assistance of multiple structures like nuclear pores.^44^ The RS model successfully explains the breakdown of nuclear mRNA buffering when the nuclear pore component NUP93 and the core components of the nuclear RNA exosome EXOSC3 and EXOSC5 are knocked down,^17^ strongly supporting that the RS model captures the key ingredients of mRNA buffering.

### The RS model leads to mRNA buffering as the cell volume increases

We then sought to test if the RS model can achieve homeostasis of mRNA concentration during cell growth (Figure 3A). For simplicity, we assumed that the total copy numbers of proteins X and Y are proportional to the cell volume, which is valid for most proteins.^45^ We first fit the experimental data of mRNA production rate vs. cell volume from ref. Swaffer et al.^26^ using Equation 13 assuming a constant ratio between the total cell volume and nuclear volume (the blue line in Figure 3B). From this fitting, we obtained the value of the MM parameter $K_{v}$ for wild-type (WT) cells (Equation 13). $K_{v}$ and other parameters inferred from the fitting of kinetic data of budding yeast (see the next section) constitute the basic parameters in this work (STAR methods and Table 1). In the above fitting, we neglected the volume dependence of the concentrations of transcription factors, $c_{Xp}$ and $c_{Yp}$, and confirmed that this approximation was valid (Figure S5). The nuclear mRNA concentration $c_{mn}$ is also constant in Equation 13 due to mRNA buffering, as shown in the following.

We defined the mRNA degradation rate $\delta_{m}$ as the degradation rate per mRNA, i.e., $\delta_{m}=\beta_{m}N_{Xc}/V_{c}$ according to Equation 4. Therefore, the mRNA lifetime $\tau_{m}=1/\delta_{m}$. Notably, the predicted mRNA degradation rate from the RS model (the red line in Figure 3B) precisely matches the experimentally measured values (red triangles) without any fitting parameters. The predicted mRNA number is proportional to cell volume (the yellow line in Figure 3B) and matches experimental data reasonably well (yellow squares). We notice the discrepancy in large cells that is beyond the RS model, which we will discuss in more details in the discussion section. Within the RS model, while the mRNA production rate scales sublinearly with the cell volume, the degradation rate decreases accordingly to achieve a constant mRNA concentration (Figure 3B). The RS model shows that the mRNA degradation rate automatically compensates for the sublinear mRNA production rate, consistent with experiments. In fact, according to the RS model, any cell volume dependence of the mRNA production rate will lead to a constant mRNA concentration. We confirmed this prediction by assuming an mRNA production rate that is a superlinear (Figure 3C) or oscillating (Figure 3D) function of cell volume. In both cases, the mRNA degradation rate self-adjusts to compensate for the mRNA production rate and generates a constant mRNA concentration. This stringent prediction can be tested experimentally.

### Slowed mRNA degradation after Pol II depletion

Swaffer et al.^26^ depleted half of Pol II in budding yeast and measured the degradation of a particular gene’s mRNA. They found that mRNA degradation slowed down after Pol II depletion. We found that this experimental observation is a direct prediction of the RS model. According to the RS model, the mRNA degradation rate should decrease if the mRNA production rate decreases. Because the Pol II copy number is proportional to the nuclear volume, reducing the Pol II concentration to its half value is equivalent to doubling the MM parameter $K_{v}$ in Equation 13. Once Pol II is depleted, the mRNA production rate immediately drops, leading to the drop of Xn, which further triggers the drop of the DF state Xc in the cytoplasm since the replenishment of Xc becomes slower (Equation 11). It is the reduced Xc level that leads to slowed mRNA degradation. For the TF state Xp, the drop in the mRNA production rate reduces its outflux, while Xc keeps shuttling back to the nucleus, leading to an accumulation of Xp in the nucleus. Because Xp is also a transcription activator, the accumulation of Xp enhanced the recruitment of free Pol II to promoters, alleviating the effect of Pol II depletion. Therefore, the mRNA production rate increases compared to the rate immediately after depletion of Pol II (although it is still lower than the rate before depletion).

To verify our idea, we numerically simulated a slightly modified model in which we separated the transcription of a single gene from the rest of the genome. We monitored the time dependence of the mRNA number of the particular gene (including both the nuclear and cytoplasmic mRNA) after turning off its transcription. We first fitted the data of mRNA production rate vs. cell volume before Pol II depletion by optimizing parameters except $K_{v}$ in the set of basic parameters (blue lines and circles in Figure 3E, and STAR methods). We then doubled $K_{v}$ to model Pol II depletion. We found that the RS model nicely predicts the slowed mRNA degradation after Pol II depletion without further fitting (red lines and triangles in Figure 3E).

### Temporal transcription dynamics after rapid degradation of protein X

**Figure 4:**
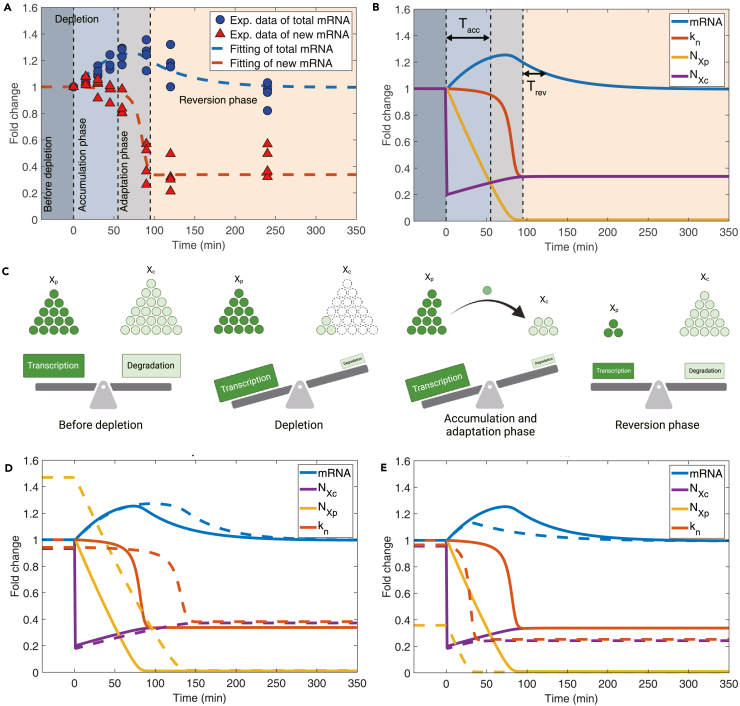
The RS model predicts the temporal change of mRNA concentration after an acute perturbation of the degradation factor Exp., experiment. (A) The temporal changes in the total mRNA concentration (total mRNA) and recently transcribed mRNA concentration (new mRNA) following acute depletion of the degradation factor X. The data points represent experimental measurements from Chappleboim et al.^14^ The dashed lines are fitting of the experimental data by optimizing parameters in the RS model ($MSE=2.10\times 10^{-3}$). We used the mRNA production rate as a proxy for the recently transcribed mRNA concentration. The fold changes represent the relative concentrations of total and new mRNA compared to those before Xrn1 depletion. (B) Simulation results of the temporal dynamics of mRNA, $k_{n}$, Xp, and Xc. The fold changes represent the relative ratios of the mRNA production rate $k_{n}$, the numbers of mRNA, Xc, and Xp, compared to those before Xrn1 depletion. (C) The schematic illustrating the dynamics of X after an acute depletion of Xc and its influence on transcription and degradation at different times. (D) Impact of a lower Pol II concentration (a larger $K_{v}$) on the temporal changes following depletion of Xc. The solid lines are WT cells, and the dashed lines are for cells with a larger $K_{v}$. The fold changes represent the relative values of the WT cells and the high-$K_{v}$ cells, compared to those in the WT cells before depletion. (E) Influence of a lower X concentration (a lower $c_{Xt}$) on the temporal changes after depleting Xc. The fold changes represent the relative values of the WT cells and the low-$c_{Xt}$ cells, compared to those in the WT cells before depletion.

Chappleboim et al.^14^ rapidly depleted Xrn1 in budding yeast and monitored the temporal dynamics of the total mRNA concentration and the level of recently transcribed mRNA, which is a good proxy for the mRNA production rate. Experimental observations revealed three phases of mRNA dynamics upon the sudden removal of the protein Xrn1 (Figure 4A). In the accumulation phase, the mRNA concentration increased, and the mRNA production rate remained almost constant. In the adaptation phase, the mRNA production rate dropped rapidly, and the mRNA concentration ceased to increase. In the reversion phase, the mRNA production rate reached the new steady-state value, and the mRNA concentration gradually reduced to its original value before the depletion of Xrn1. To verify whether the RS model can explain the temporal dynamics of mRNA as a more stringent test, we studied the RS model after a rapid depletion of protein X.

First, we remark that, in the RS model, protein X is presumably a coarse-grained combination of several proteins. This idea is supported by the experimental observations that mRNA buffering was still valid even if Xrn1 was knocked out,^8^^,^^14^ which we discuss in detail later. Therefore, the depletion of Xrn1 should correspond to a partial depletion of protein X in the RS model. Because Xrn1 is the primary degradation factor in budding yeast^8^ and more than 90 percent copies of Xrn1 are localized in the cytoplasm,^46^ we modeled the depletion of Xrn1 as a rapid reduction of $N_{Xc}$ to its 20% value in the cytoplasm. We neglected the depletion of protein X in the nucleus, including the TF state Xp and the released state Xn for simplicity. This assumption can be relaxed as long as the nuclear X does not decrease significantly.

Due to the rapid depletion of the DF state Xc in the cytoplasm, the replenishment of Xp (Equation 6) significantly decreases (Figure 4B). However, we argue that a delay between the decrease of the mRNA production rate and the reduction of Xp can emerge because the transcription factor concentration is still much higher than the MM constant $K_{x}$ (Equation 13). In this case, the mRNA production rate will be maintained at a constant value for a finite duration, in agreement with experiments.^14^ Therefore, the TF state Xp initially decreases linearly because of the conversion from Xp to Xn (Equation 6). The linear reduction of Xp holds until $N_{Xp}/V_{n}$ hits $K_{x}$, which leads to a significant decrease in the mRNA production rate (Equation 13) (Figure S6). Therefore, we can approximate the duration of the accumulation phase as $T_{acc}\approx \left(N_{Xp}-K_{x}V_{n}\right)/\left(k_{n,bf}f_{dep}\right)$ according to Equation 6. Here, $k_{n,bf}$ is the mRNA production rate before perturbation, which is also the replenishment rate from Xc to Xp. We have taken account of the reduced replenishment rate as $\left(1-f_{dep}\right)k_{n,bf}$ where $f_{dep}$ is the fraction of depleted Xc (80% in this case). The values of $N_{Xc}$, $N_{Xp}$, and the mRNA production rate then quickly reach the new steady-state values, after which the mRNA concentration gradually returns to its original value with a relaxation time determined by the mRNA degradation rate, $\delta_{m}=\beta_{m}N_{Xc}/V_{c}$ according to Equation 4. Therefore, the duration of the reversion phase is $T_{rev}\approx V_{c}/\beta_{m}N_{Xc}$. Figure 4C shows a schematic of this process.

In the earlier discussion, the initial value of the transcription factor concentration $c_{Xp}$ must be far above the parameter $K_{x}$ (Figure S6). This condition suggests that for WT cells, Xp is generally non-limiting for transcription (although it is still necessary to initiate transcription). We also confirmed that the three phases in the transcription dynamics following an acute depletion are robust against different choices of the parameters (Figure S7). In conclusion, the predictions of the RS model not only perfectly aligned with the experimental data from which we inferred the set of basic parameters (Figure 4A and STAR methods) but also provided valuable insights into the underlying mechanism (Figures 4B and 4C).

Next, we sought to systematically investigate the transcription dynamics after acute depletion of Xc according to the RS model, in particular, considering mutant cells with different concentrations of Pol II or protein X. Future experiments can test our predictions. We compared a WT cell (solid lines in Figure 4D) and a mutant cell with a lower Pol II concentration (dashed lines in Figure 4D). The mutant with a lower Pol II concentration, equivalent to a larger $K_{v}$ in the RS model (Equation 13), has a mildly lower mRNA production rate than the WT cell. Therefore, the mutant has fewer Xn and Xc compared to the WT cell (Equations 10 and 11), leading to a higher copy number of Xp in the mutant due to the conservation of total number of protein X. Consequently, it takes longer for the mutant cell to reach the adaptation phase since it needs a longer time for the concentration of Xp, $c_{Xp}$, to decrease to the MM constant $K_{x}$ after which $k_{n}$ starts to drop quickly, followed by the decrease of mRNA copy number. Because of the longer duration of the accumulation phase for the similar accumulation rate of Xc between the mutant and the WT cell, the mutant has a higher Xc in the new steady state. Therefore, the mutant recovers more rapidly during the reversion phase than the WT cell.

For the mutant (dashed lines in Figure 4E) with a lower protein X concentration, it has fewer TF state Xp than the WT cell (solid lines in Figure 4E). However, its mRNA production rate remains close to the WT cell as long as the concentration of Xp is still significantly larger than $K_{x}$. Because of the fewer Xp, the mutant exhibits a shorter accumulation phase than the WT cell because the duration of the accumulation phase depends on the initial $N_{Xp}$, $T_{acc}\approx \left(N_{Xp}-K_{x}V_{n}\right)/\left(k_{n,bf}f_{dep}\right)$. Therefore, the mutant also has a lower number of Xc during the reversion phase, so the mRNA concentration recovers slower in the mutant than in the WT cell. We also investigated the temporal transcription dynamics for other mutants with different modified parameters (Figure S7).

### Candidates of X and Y

**Figure 5:**
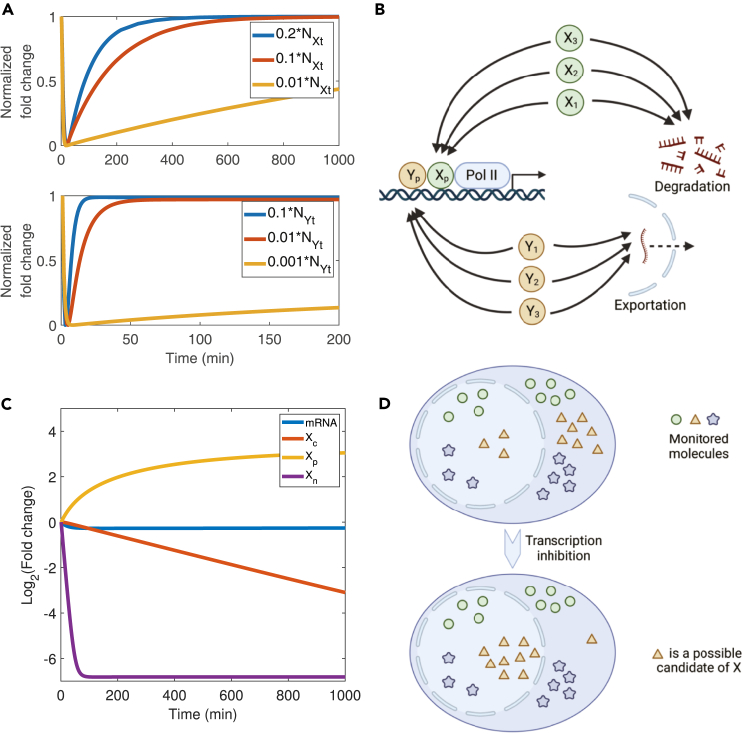
Proteins X and Y should represent groups of proteins with similar functions (A) The recovery of cytoplasmic mRNA (upper panel with varying copy numbers of X) and nuclear mRNA (lower panel with varying copy numbers of Y) after a rapid perturbation to the mRNA production rate. The y axis normalized fold change is shifted and normalized so that the value before perturbation is one and the minimum value is 0. (B) Schematic illustrating X and Y can be groups of different proteins executing similar functions. (C) The time course of the mRNA concentration, $N_{Xc}$, $N_{Xp}$, and $N_{Xn}$ after the complete shutoff of transcription. The fold changes represent the relative values of the perturbed cells compared to those before the transcription shutoff. (D) Protein X in the cytoplasm will gradually shuttle to the nucleus until most of its copies are localized in the nucleus after a complete transcription inhibition, which can be used to detect the candidates of protein X.

Experiments showed that the mRNA concentration was buffered even when the global degradation factors such as Dcp2 and Xrn1 were knocked out.^8^^,^^14^ These factors play vital roles in 5′-3′ mRNA degradation, the predominant pathway of mRNA degradation.^1^ These observations suggest that the protein X and Y in the RS model may not represent a single protein, and they may be a combination of multiple proteins. To demonstrate this idea explicitly, we investigated the response of mRNA concentration to an abrupt drop in the mRNA production rate by considering cells with different total numbers of protein X. We found that the recovery time of mRNA concentration diverges in the limit of $N_{Xt}\to 0$ (upper panel of Figure 5A). This result suggests that, if protein X is only Xrn1, cells with Xrn1 knocked out should not recover from a slight noise in the mRNA production rate, so it cannot achieve mRNA buffering, contradicting experiments. We analyzed protein Y similarly and obtained the same results (lower panel of Figure 5A). Therefore, we proposed that X and Y represent groups of multiple proteins that perform redundant functions (Figure 5B).

The necessity of the two transcription factors, Xp and Yp, to transcription (Figure S2) implies that they must be essential components of the transcription machinery, such as the subunit of Pol II, the general transcription factors, and other necessary molecules. Rpb4 and Rpb7, the subunits of Pol II, are possible candidates involved in shuttling across the nuclear membrane and regulating mRNA degradation.^4^^,^^31^^,^^32^^,^^33^ Although the cytoplasmic function of Rpb4 is shown to be dispensable,^47^ similar functions may exist in other factors of the transcription machinery. CDK8, orthologous to budding yeast Ssn3, is a cyclin-dependent kinase (CDK) functional in phosphorylating the CTD of Pol II, promoting the assembly of the elongation complex.^48^ Evidence shows that CDK8 participates in the degradation of several mRNAs related to metabolism.^49^^,^^50^ Hmt1 is a methyltransferase in budding yeast, which participates at the beginning of the transcriptional elongation process and influences mRNA export by methylating some RNA-binding proteins (RBPs).^51^ SUS1 encodes a protein that is a shared subunit of two complexes: Spt-Ada-Gcn5 acetyltransferase (SAGA) and transcription and export complex 2 (TREX-2). SAGA modifies chromatin structure and facilitates transcription initiation, while TREX-2 is a complex that associates with the nuclear pore complex and mediates mRNA export.^52^ In a nutshell, we provided a list of candidates of X and Y, participating in at least two of the three processes, including transcription, export, and degradation (STAR methods and Table S1).

Typically, genetic perturbation methods map a gene to a phenotype of interest. If the knockout or knockdown of a particular gene affects the phenotype, it suggests that the gene is likely responsible for the phenotype. However, in the case of protein X, presumably a combination of proteins with redundant functions, perturbing a single gene may not significantly impact mRNA buffering, making it challenging to identify the candidates responsible for mRNA buffering using traditional approaches. Fortunately, the RS model suggests an alternative solution. We found that the RS model made an interesting prediction if transcription is completely inhibited, e.g., through rapid Pol II depletion or transcription-inhibiting drugs. In this case, the transformation from the TF state Xp to the released state Xn is blocked, and Xn quickly reduces to zero. Meanwhile, the DF state Xc in the cytoplasm gradually shuttles back to the nucleus to become Xp (Figure 5C). During this process, mRNA continues to be degraded and eventually stabilizes at a lower level (Figure 5C). By monitoring the cytoplasmic proteins that are predominantly transported back to the nucleus after transcription shutoff, one can identify the possible constituents of X (Figure 5D). In this scenario, possible candidates of protein X, e.g., Xrn1, Rpb4/7, etc., are predicted to accumulate in the nucleus. This prediction of the RS model provides an experimental protocol to examine the potential candidates for protein X. We acknowledge that real-world scenarios may deviate from these predictions, and measuring concentration differences between the nucleus and cytoplasm can be technically challenging. Further experimental data are necessary to refine and modify the RS model accordingly.

### Extensions of the RS model

The RS model provides a fundamental understanding of transcription regulation by revealing the coordination of mRNA production, export, and degradation rates to maintain a constant mRNA concentration. However, it is essential to recognize that the real-world dynamics of mRNA regulation can be more complex than the current RS model. This section discusses some of the complexities that may go beyond the simplified scenario of mRNA buffering and how the RS model can be extended to address them.

We first discuss the mRNA dynamics during viral infection. When cells are infected, the mRNA degradation rates are enhanced to facilitate the rapid turnover of host mRNA and redirect cellular resources toward viral replication, a phenomenon called “host shutoff.”^53^ Contrary to mRNA buffering, the mRNA production rate becomes slower, although the mRNA degradation rate is accelerated by viral infection.^54^ Recently, Gilbertson et al. showed that in mammalian cells, the poly(A)-binding protein cytoplasmic 1 (PABPC1) plays a critical role in this process.^36^ PABPC1 is an RBP that binds directly to the poly(A) tail of the pre-mRNA in the nucleus and is released in the cytoplasm after mRNA is degraded, functioning in mRNA translation and degradation.^55^^,^^56^^,^^57^ Researchers found that it also interferes with the formation of PIC in the nucleus.^36^ Once the viral endonuclease accelerates mRNA degradation, more PABPC1 proteins are released into the cytoplasm, which then shuttle to the nucleus and inhibit transcription.^36^

**Figure 6:**
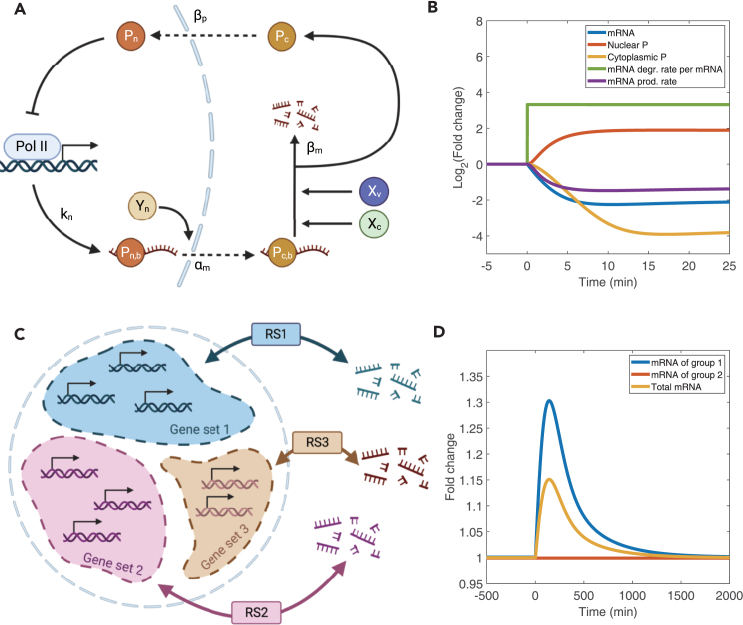
The extensions of the RS model Degr., degradation; prod., production. (A) Schematic of the modified model in which a third protein P is added. $X_{v}$ represents the viral endonuclease that increases the factor $\beta_{m}$. (B) Simulations of the dynamics of the total mRNA concentration, nuclear P ($N_{Pn}+N_{Pnb}$), cytoplasmic P ($N_{Pc}+N_{Pcb}$), degradation rate per mRNA, and production rate after a sudden increment of $\beta_{m}$ at time 0. The fold changes represent the relative values of the perturbed cells compared to those before the sudden increment of $\beta_{m}$. (C) Schematic of the generalized model in which different sets of genes are regulated by different groups of X and Y, e.g., RS1, to buffer mRNA concentration separately. (D) Simulations of the dynamics of two groups of mRNA regulated by distinct groups of X and Y and their sum after a sudden decrement in Xc of group 1 at time 0. The fold changes represent the relative mRNA numbers of the perturbed cells compared to those before the perturbation.

With this experimental evidence, we modified the RS model by adding a third protein P representing PABPC1 (Figure 6A and STAR methods). In this modified model, there are four states of P, including the one binding to the nuclear mRNA (Pnb), the one binding to the cytoplasmic mRNA (Pcb), the free P in the nucleus (Pn), and the free P in the cytoplasm (Pc). We assumed that protein P binds mRNA tightly and gets released only when the mRNA is degraded so that the numbers of Pnb and Pcb are equal to the nuclear mRNA and cytoplasmic mRNA, respectively. Pnb is assumed to be exported along with mRNA, while the rate of Pc shuttling into the nucleus is determined by the factor $\beta_{p}$. We also modeled the inhibitions of Pn to the mRNA production rate as an MM function. We modeled the effects of viral infection as an increased mRNA degradation rate by increasing $\beta_{m}$ (Equation 4). Simulations showed that, upon viral infection, the mRNA degradation rate per mRNA increases while the mRNA production rate decreases so that the mRNA concentration decreases (Figure 6B, and see STAR methods for the details of the simulations). In the meantime, protein P is enriched in the nucleus, in agreement with experimental observations during viral infection.

Next, we discuss the differential regulations of specific mRNA groups.^1^^,^^14^^,^^29^^,^^58^ Recently, Chattopadhyay et al. showed that blocking the import of Xrn1 to the nucleus significantly reduces the production and degradation rate of a subset of mRNAs but does not affect the rest.^29^ Another example is that the expression of ribosomal genes responds differentially to the knockout of Ccr4-Not and Xrn1.^58^ Interestingly, in both cases, mRNAs related to ribosome biogenesis appear to be regulated distinctly. Given these experimental observations, we propose that separate sets of proteins X and Y can coexist to buffer each group of mRNA independently (Figure 6C), contributing to the overall homeostasis of mRNA concentration. One benefit of separate sets of proteins X and Y is that the expression changes of one group of genes do not significantly influence the other groups. To demonstrate this benefit explicitly, we modified the RS model by introducing two sets of X and Y, regulating two groups of genes. Simulations of this modified RS model show that, despite changes in the mRNA concentration in one group after perturbing its Xc, the mRNA concentration of the other group is largely invariant (Figure 6D, see STAR methods for the details of the simulations).

Previous experiments have suggested that the catalytic subunit Rrp6 of the nuclear exosome participates in the regulation of mRNA levels, indicating a potential effect of the nuclear degradation pathways.^9^^,^^59^^,^^60^ Therefore, we studied an extended model in which we incorporated nuclear degradation by introducing a new protein Z into the current model (STAR methods and Figure S8A). In this modified model, the exosome is both a nuclear decay factor and a transcription factor. We found that mRNA buffering is still valid in this modified model (Figure S8B).

The current form of the RS model does not require persistent binding between mRNAs and proteins X and Y. However, the mRNA imprinting phenomenon, in which some decay factors bind to and shuttle along with mRNA, is suggested to function in mRNA buffering.^33^ Mathematical models of mRNA imprinting have also been built in which the crosstalk factors impact transcription and mRNA stability.^35^ To test whether our model can achieve mRNA buffering with imprinting, we introduced a modified model in which Xn binds to newly produced mRNA after transcription and shuttles out of the nucleus along with that mRNA (STAR methods and Figure S9A). Interestingly, our conclusions regarding mRNA buffering and the transient behaviors after Xrn1 depletion remain the same (Figure S9B).

In real-world transcription, the drop-off phenomenon (also known as premature transcription termination) may occur, in which Pol II stops transcription prematurely.^61^ To check the validity of our model, we introduced a global constant drop-off probability to an extended model and found that mRNA buffering still holds (STAR methods).

In summary, while the RS model provides a powerful framework for understanding mRNA buffering, its simplicity also allows for straightforward extensions and adaptations to capture the complexity of mRNA regulation. By incorporating additional regulatory information, the RS model can be tailored to address specific biological contexts and shed light on the diverse regulation mechanisms of the mRNA level.

## Discussion

In this study, we introduce the RS model as a mechanistic framework to explain mRNA buffering, a fascinating phenomenon observed in various organisms where mRNA concentrations remain approximately constant through coupled changes in mRNA production and degradation rates. The RS model incorporates two essential proteins, X and Y, involved in transcription, export, and degradation. The model demonstrates that the clue of the mRNA production rate is conveyed downstream via X and Y, synchronizing all rates to achieve mRNA buffering. Through mathematical modeling and analysis, the RS model provides a robust mechanism of mRNA buffering and makes valuable predictions that are experimentally testable. We demonstrated that feedback loops from degradation to transcription are neither necessary nor able to explain mRNA buffering (Figure S1). Instead, as long as a DF state of protein X and an EF state of protein Y are released after each transcription initiation, the mRNA degradation rate, the mRNA export rate, and the mRNA production rate are automatically balanced such that the nuclear and cytoplasmic mRNA concentrations are independent of the mRNA production rate.

We remark that, at the single-cell level, effects from different cellular processes may significantly influence the homeostasis of mRNA concentration. Mathematical models have explored mRNA concentration homeostasis at the single-gene and single-cell level, considering various biological processes, such as cell growth and DNA replication, connecting single-cell gene expression data to the underlying gene expression dynamics.^62^^,^^63^^,^^64^^,^^65^ The RS model, on the other hand, mainly focuses on the homeostasis of mRNA concentration at the bulk level against various perturbations, e.g., gene knockout. While we illustrate the molecular pathways of the RS model using the picture of a single cell, this work aims to study the mRNA dynamics at the bulk level. Thus, our model should be considered as a model of gene expression dynamics for an averaged cell over a population.

mRNA splicing is another essential process during mRNA metabolism.^66^ It plays a crucial role in mRNA homeostasis, as proper processing and packaging within the nucleus are prerequisites for mRNA export to the cytoplasm. We remark that the current RS model does not explicitly include splicing, and splicing factors may also be candidates of X and Y.

### Limitations of the study

While the RS model successfully explains mRNA buffering and makes valuable predictions, it also shows limitations and raises opportunities for further investigation. First, we noticed a tendency for mRNA concentrations to decrease in large cells (Figure 3B).^26^ In more extreme experiments, the mRNA and protein concentrations decreased significantly in budding yeast cells with extremely large sizes.^67^ The RS model by itself does not include the dynamics of cell volume. We think that the decrease of mRNA concentration in giant cells may be due to the imbalance of biomass production and osmolyte synthesis,^68^^,^^69^ beyond the scope of the RS model. The impact of cell volume on mRNA homeostasis is crucial and warrants consideration. Future extensions of the RS model include coupling cell volume and gene expression dynamics to capture better the interplay between mRNA production, degradation, and cell growth. Second, Chattopadhyay et al.^29^ mentioned that the total mRNA concentration is unaffected by mutations in the nuclear localization sequences of Xrn1. However, the RS model predicts a lower mRNA concentration if Xrn1, as a global decay factor of mRNA, is retained in the cytoplasm, which mathematically corresponds to a smaller $\beta_{x}$ in Equation 9. In summary, further quantitative data and theoretical analysis are needed to guide the modification of the RS model to incorporate other critical biological features.

## STAR★Methods

### Key resources table

**Table:** 

REAGENT or RESOURCE	SOURCE	IDENTIFIER
**Deposited data**		

The data of the mRNA production rates with global genetic perturbations in mammalian cells.	Berry et al.^17^	Mendeley Data: https://doi.org/10.17632/3v4bkmg92x.1
The data of the total mRNA with global genetic perturbations in mammalian cells.	Berry et al.^17^	Mendeley Data: https://doi.org/10.17632/yfx32prktv.1
The data of the mRNA production and degradation rates with global genetic perturbations in budding yeast cells.	Sun et al.^9^	Molecular Cell: https://doi.org/10.1016/j.molcel.2013.09.010
The data of the mRNA production and degradation rates and the mRNA amount in budding yeast cells with different volumes.	Swaffer et al.^26^	Cell: https://doi.org/10.1016/j.cell.2023.10.012
The data of the mRNA degradation after perturbing Pol II in budding yeast cells.	Swaffer et al.^26^	Cell: https://doi.org/10.1016/j.cell.2023.10.012
The data of the mRNA dynamics after acute depletion of Xrn1.	Chappleboim et al.^14^	Zenodo: https://zenodo.org/doi/10.5281/zenodo.6528287

**Software and algorithms**		

Original codes for mathematical simulations.	This paper	Github: https://github.com/QirunWang/Codes-for-the-RS-model

### Resource availability

#### Lead contact

Further information and requests for resources should be directed to and will be fulfilled by the lead contact, Jie Lin (linjie@pku.edu.cn).

#### Materials availability

This study did not generate new biological materials.

#### Data and code availability

• This paper analyzes existing, publicly available data, which are summarized in the key resources table. The experimental data of the mRNA production rates as well as the total mRNA with global genetic perturbations in mammalian cells were obtained from.^17^ The experimental data of the mRNA production and degradation rates with global genetic perturbations in budding yeast cells were obtained from.^9^ The fold changes of the mRNA production rate (i.e., Pol II occupancy) and the mRNA amount in cells with different volumes were obtained from Figure 6A of,^26^ while the mRNA degradation rates under volume changes were obtained from Figure S8B of.^26^ The experimental data of perturbing Pol II were obtained from Figure S8E of.^26^ The experimental data the mRNA dynamics after acute depletion of Xrn1 were obtained from.^14^.
• All original codes for mathematical simulations are available in the following link (https://github.com/QirunWang/Codes-for-the-RS-model).
• Any additional information required to reanalyze the data reported in this paper is available from the lead contact upon request.

### Method details

#### Details of the feedback models

In the positive feedback model where mRNA activates its own degradation (Figure S1A), the dynamics of mRNA concentration follows

(Equation 14) $$\frac{dc_{m}}{dt}=k_{n}-\beta_{m}c_{m}^{2}$$

where $k_{n}$ is the production rate, and $\beta_{m}c_{m}^{2}$ is the degradation rate, a nonlinear mRNA concentration function. We sampled $k_{n}$ uniformly within a finite range and monitored the relationship between the mRNA production rate $k_{n}$ and the steady-state mRNA concentration $c_{m}$ (Figure S1B). We also sampled $\beta_{m}$ uniformly within a finite range and monitored the relationship between $k_{n}$ and $c_{m}$ (Figure S1C). We randomly sampled the mRNA concentrations from a Poisson distribution with the means equal to the steady-state values derived from the positive feedback model, mimicking noises in gene expression. We also added Gaussian noise on top of the mRNA production rates so that the CVs equal 0.003. In neither case we observed mRNA buffering.

In the negative feedback model where mRNA inhibits its own production (Figure S1D), the dynamics of mRNA concentration follows

(Equation 15) $$\frac{dc_{m}}{dt}=\frac{a}{c_{m}+b}-\beta_{m}c_{m}\text{,}$$

where *a* and *b* are constants. We sampled *a* uniformly within a finite range and monitored the relationship between $k_{n}$ and $c_{m}$ (Figure S1E). We also sampled $\beta_{m}$ uniformly within a finite range and monitored the relationship between $k_{n}$ and $c_{m}$ (Figure S1F). We randomly sampled the mRNA concentrations from a Poisson distribution with the means equal to the steady-state values derived from the negative feedback model, mimicking noises in gene expression. We also added Gaussian noise on top of the mRNA production rates so that the CVs equal 0.003. In neither case we observed mRNA buffering.

#### The modified models in which X and Y are not necessary for transcription

We assumed that transcription is accelerated by the binding of Xp to the preinitiation complex (PIC), and transcription can also proceed without Xp (we still kept the necessity of Yp to transcription for simplicity). We assumed that Xp binds to the PIC and hops off with a binding affinity $K_{x}$. PICs bound to Xp initiate transcription faster than PICs unbound to Xp. Therefore, the mRNA production rate can be decomposed into two parts

(Equation 16) $$k_{n,0}=k_{1}\frac{K_{x}}{K_{x}+c_{Xp}}\text{,}$$

(Equation 17) $$k_{n,p}=k_{2}\frac{c_{Xp}}{K_{x}+c_{Xp}}\text{,}$$

where $k_{1}$ and $k_{2}$ are coarse-grained variables, including the other factors in Equation 13 of the maintext, i.e., $k_{1}=k_{0,1}\frac{V_{n}}{V_{n}+K_{v}}\frac{K_{m}}{c_{mn}+K_{m}}\frac{c_{Yp}}{c_{Yp}+K_{y}}$ and $k_{2}=k_{0,2}\frac{V_{n}}{V_{n}+K_{v}}\frac{K_{m}}{c_{mn}+K_{m}}\frac{c_{Yp}}{c_{Yp}+K_{y}}$, where $k_{0,1}<k_{0,2}$. Here, $c_{Xp}=N_{Xp}/V_{n}$ is the concentration of Xp in the nucleus. The term $\frac{c_{Xp}}{K_{x}+c_{Xp}}$ in Equation 17 is the probability of a PIC bound by Xp. Therefore, the term $\frac{K_{x}}{K_{x}+c_{Xp}}$ in Equation 16 is simply the probability of a PIC not bound by Xp.

The total mRNA production rate $k_{n}=k_{n,0}+k_{n,p}$, while the transformation rate form Xp to Xn is $k_{n,p}$. We rewrote Equations 1, 2, 3, 4, 5, 6, and 7 as

(Equation 18) $$\frac{dN_{mn}}{dt}=k_{n,0}+k_{n,p}-\alpha_{m}\frac{N_{Yn}}{V_{n}}N_{mn}\text{,}$$

(Equation 19) $$\frac{dN_{mc}}{dt}=\alpha_{m}\frac{N_{Yn}}{V_{n}}N_{mn}-\beta_{m}\frac{N_{Xc}}{V_{c}}N_{mc}\text{,}$$

(Equation 20) $$\frac{dN_{Xn}}{dt}=k_{n,p}-\alpha_{x}N_{Xn}\text{,}$$

(Equation 21) $$\frac{dN_{Yn}}{dt}=k_{n,0}+k_{n,p}-\alpha_{y}N_{Yn}\text{,}$$

(Equation 22) $$\frac{dN_{Xc}}{dt}=\alpha_{x}N_{Xn}-\beta_{x}N_{Xc}\text{,}$$

(Equation 23) $$\frac{dN_{Xp}}{dt}=\beta_{x}N_{Xc}-k_{n,p}\text{,}$$

(Equation 24) $$\frac{dN_{Yp}}{dt}=\alpha_{y}N_{Yn}-k_{n,0}-k_{n,p}\text{.}$$

Meanwhile, $k_{n,p}=\beta_{x}N_{Xc}$ in the steady state to keep a constant number of $N_{Xp}$. Therefore, the steady-state solution of the cytoplasmic mRNA concentration becomes

(Equation 25) $$c_{mc}=\frac{\beta_{x}}{\beta_{m}}\frac{k_{1}K_{x}V_{n}+k_{2}N_{Xp}}{k_{2}N_{Xp}}\text{.}$$

In this modified model, the cytoplasmic mRNA concentration depends on the cell volume and the copy number of $N_{Xp}$: the mRNA buffering breaks down. Numerical simulations confirmed our predictions (Figure S2). A similar analysis also applies to Y. In conclusion, the validity of mRNA buffering requires that proteins X and Y must be necessary factors for transcription initiation.

#### The modified model in which Y shuttles

In this modified model, the dynamics of protein X is the same as in the original model, while the dynamics of Y follows

(Equation 26) $$\frac{dN_{Yn}}{dt}=k_{n}-\alpha_{y}N_{Yn}\text{,}$$

(Equation 27) $$\frac{dN_{Yc}}{dt}=\alpha_{y}N_{Yn}-\beta_{y}N_{Yc}\text{,}$$

(Equation 28) $$\frac{dN_{Yp}}{dt}=\beta_{y}N_{Yc}-k_{n}\text{,}$$

where $N_{Yc}$ is the cytoplasmic Y. In this modified model, mRNA buffering is still valid (Figure S3).

#### The modified model in which the exosome participates in mRNA buffering

In this modified model, we incorporate the nuclear exosome Z as a third factor participating mRNA buffering (Figure S8A). Similar to Y, Z can be in two states: the decay factor Zn who degrades nuclear mRNA, and the transcription factor Zp who functions in transcription initiation. The dynamics of Z are

(Equation 29) $$\frac{dN_{Zn}}{dt}=k_{n}-\alpha_{z}N_{Zn}\text{,}$$

(Equation 30) $$\frac{dN_{Zp}}{dt}=\alpha_{z}N_{Zn}-k_{n}\text{,}$$

where $\alpha_{z}$ is the transformation rate from Zn to Zp. Considering nuclear degradation, the dynamics of the nuclear mRNA (Equation 1) can be written as

(Equation 31) $$\frac{dN_{mn}}{dt}=k_{n}-\alpha_{m}\frac{N_{Yn}}{V_{n}}N_{mn}-\alpha_{d}\frac{N_{Zn}}{V_{n}}N_{mn}\text{,}$$

where $\alpha_{d}$ is the degradation rate in the nucleus. Because Z also functions in transcription initiation, the transcription rate (Equation 13) can be written as

(Equation 32) $$k_{n}=k_{0}\frac{V_{n}}{V_{n}+K_{v}}\frac{K_{m}}{c_{mn}+K_{m}}\frac{c_{Xp}}{c_{Xp}+K_{x}}\frac{c_{Yp}}{c_{Yp}+K_{y}}\frac{c_{Zp}}{c_{Zp}+K_{z}}\text{.}$$

where $c_{Zp}=N_{Zp}/V_{n}$ is the concentration of Zp, and $K_{z}$ is the Michaelis-Menten constant. All other aspects of the system unchanged, which are illustrated in Equations 2, 3, 4, 5, 6, and 7. It is straightforward to find the steady-state solution of all factors:

(Equation 33) $$N_{mn}=\frac{\alpha_{z}\alpha_{y}}{\alpha_{m}\alpha_{z}+\alpha_{d}\alpha_{y}}V_{n}\text{,}$$

(Equation 34) $$N_{mc}=\frac{\beta_{x}}{\beta_{m}}V_{c}\text{,}$$

(Equation 35) $$N_{Xn}=\frac{k_{n}}{\alpha_{x}}\text{,}$$

(Equation 36) $$N_{Xc}=\frac{k_{n}}{\beta_{x}}\text{,}$$

(Equation 37) $$N_{Yn}=\frac{k_{n}}{\alpha_{y}}\text{,}$$

(Equation 38) $$N_{Zn}=\frac{k_{n}}{\alpha_{z}}\text{.}$$

We can see that mRNA buffering is still valid in this scenario (Figure S8).

#### The modified model in which transcription drop-off occurs

In this modified model, we introduced the transcription drop-off (also known as premature transcription termination), in which some Pol II molecules drop off the DNA strand during elongation. We assumed a constant global drop-off probability δ such that the mRNA production rate effectively becomes $\left(1-\delta \right)k_{n}$. Nevertheless, the production rates of Xn and Yn remain $k_{n}$ since they are released after initiation. Consequently, we arrived at the following revised equation for $N_{mn}$:

(Equation 39) $$\frac{dN_{mn}}{dt}=\left(1-\delta \right)k_{n}-\alpha_{m}\frac{N_{Yn}}{V_{n}}N_{mn}\text{.}$$

Considering steady-state, we found that

(Equation 40) $$N_{mn}=\left(1-\delta \right)\frac{\alpha_{y}}{\alpha_{m}}V_{n}\text{,}$$

(Equation 41) $$N_{mc}=\left(1-\delta \right)\frac{\beta_{x}}{\beta_{m}}V_{c}\text{,}$$

indicating constant nuclear and cytoplasmic mRNA concentrations independent of the mRNA production rate, affirming mRNA buffering.

#### The modified model in which imprinting occurs

In this modified model, we consider the effect of mRNA imprinting (namely, X binds on and shuttles along with mRNA) (Figure S9A). In this circumstance, Xn must bind to the newly produced mRNA to be exported to the cytoplasm. Therefore, the dynamics of Xn and Xc (Equations 2 and 5) are rewritten as

(Equation 42) $$\frac{dN_{Xn}}{dt}=k_{n}-\alpha_{m}\frac{N_{Yn}}{V_{n}}N_{mn}\text{.}$$

(Equation 43) $$\frac{dN_{Xc}}{dt}=\alpha_{m}\frac{N_{Yn}}{V_{n}}N_{mn}-\beta_{x}N_{Xc}\text{.}$$

With all other dynamics of the system (Equations 1, 3, 4, 6, and 7) unchanged, we found the steady-state solution of all factors:

(Equation 44) $$N_{mn}=N_{Xn}=\frac{\alpha_{y}}{\alpha_{m}}V_{n}\text{,}$$

(Equation 45) $$N_{mc}=\frac{\beta_{x}}{\beta_{m}}V_{c}\text{,}$$

(Equation 46) $$N_{Xc}=\frac{k_{n}}{\beta_{x}}\text{,}$$

(Equation 47) $$N_{Yn}=\frac{k_{n}}{\alpha_{y}}\text{.}$$

It is straightforward to see that the concentrations $N_{mn}/V_{n}$ and $N_{mc}/V_{c}$ are still independent of the mRNA production rate, i.e., mRNA buffering still holds. In addition, we showed that the dynamics of the modified model after acute depletion of X also agree with the experimental observations (Figure S9B).

#### The modified model for the viral infection

To explain the experimental observations of the viral infection, we added another protein, P, to the RS model, representing protein PABPC1 (Figure 6A). The dynamics of P follows

(Equation 48) $$\frac{dN_{Pnb}}{dt}=k_{n}-\alpha_{m}\frac{N_{Yn}}{V_{n}}N_{Pnb}\text{,}$$

(Equation 49) $$\frac{dN_{Pcb}}{dt}=\alpha_{m}\frac{N_{Yn}}{V_{n}}N_{Pnb}-\beta_{m}\frac{N_{Xc}}{V_{c}}N_{Pcb}\text{,}$$

(Equation 50) $$\frac{dN_{Pc}}{dt}=\beta_{m}\frac{N_{Xc}}{V_{c}}N_{Pcb}-\beta_{p}N_{Pc}\text{,}$$

(Equation 51) $$\frac{dN_{Pn}}{dt}=\beta_{p}N_{Pc}-k_{n}\text{.}$$

Here $N_{Pnb}=N_{mn}$ and $N_{Pcb}=N_{mc}$ because P binds to mRNA tightly. To model the inhibition of PABPC1 to transcription, we included the concentration of free protein Pn to Equation 13, so that

(Equation 52) $$k_{n}=k_{0}\frac{V_{n}}{V_{n}+K_{v}}\frac{c_{Xp}}{c_{Xp}+K_{x}}\frac{c_{Yp}}{c_{Yp}+K_{y}}\frac{K_{m}}{c_{mn}+K_{m}}\frac{K_{p}}{c_{Pn}+K_{p}}\text{,}$$

where $c_{Pn}$ is the concentration of Pn in nucleus, and $K_{p}$ is a constant.

#### Finding candidates of X and Y

The RS model suggests that X and Y are multifunctional molecules involved in transcription, export, and degradation. To identify candidate genes in these categories, we utilized various databases, including the Gene Ontology (GO) database through AmiGO (version 2.5.17),^70^^,^^71^^,^^72^ the Kyoto Encyclopedia of Genes and Genomes (KEGG) database,^73^^,^^74^^,^^75^ and the Saccharomyces Genome Database (SGD).^76^ In our search for transcription-related genes, we focused on those annotated as transcription factors or involved in the regulation of transcription, as well as the subunits of Pol II. For export-related genes, we looked for annotations related to nuclear transportation factors and the subunits of the nuclear pore. In the context of degradation, we identified genes involved in mRNA catabolism. We marked genes with dual functions in transcription and export as Y and genes with dual functions in transcription and degradation as X (Table S1).

#### Analysis of experimental data

In Figures 2A and 2B, the experimental data were obtained from.^17^ The nuclear mRNA concentrations, cytoplasmic mRNA concentrations, and mRNA production rates were calculated by averaging the “mean nucleus mean FISH,” “mean cytoplasm mean FISH,” and “mean nucleus mean EU” values, respectively, across all samples perturbing the same gene (or scrambled pieces). The data were then normalized by the mean values of the scrambled samples to obtain fold changes.

In Figure 3E, the experimental data for WT cells and cells with half of the Pol II depleted were obtained from Figure S8E of.^26^ We set the time point at 6 min as the starting time point, the values of which were set to 1. Only the data after the starting time point were used. We then fitted the data of WT cells with a one-phase exponential decay model to get the degradation rates per mRNA ($r^{2}=0.97$).

In Figure 4A, the experimental data were obtained from the supplementary table “xrn1 global nas traj repeats” in.^14^ mRNA levels were obtained from “smoothed mRNA”, and newly transcribed mRNA levels were obtained from “nascent”. All values were normalized with the corresponding values at time 0 to obtain fold changes.

#### Determination of basic parameters

In the RS model, there are a total of 14 unknown parameters: $k_{0}$, $K_{x}$, $K_{y}$, $K_{m}$, $K_{v}$, $\alpha_{m}$, $\alpha_{x}$, $\alpha_{y}$, $\beta_{m}$, $\beta_{x}$, $N_{Xt}$, $N_{Yt}$, $V_{n}$, and $V_{c}$. Exploring the entire parameter space to find combinations that quantitatively fit the experimental data is a complex task due to the large number of parameters involved. Therefore, we fixed some parameters within biologically reasonable ranges to simplify the analysis and made some reasonable assumptions. More quantitative experiments are necessary to refine the parameters and improve our understanding of the RS model in the future.

We assumed a total cell volume of 40 fL, the typical volume of haploid *S. Saccharomyces*. Since the nuclear volume is approximately 7% of the total cell volume,^77^ we set the nuclear volume ($V_{n}$) to 2.8 fL and the cytoplasmic volume ($V_{c}$) to 37.2 fL. Throughout the simulations, the values of $V_{n}$ and $V_{c}$ are kept fixed, except for the simulations involving cell growth (Figures 3B, 3C, and 3D), where the ratio between $V_{n}$ and $V_{c}$ is maintained while the cell volume increases.

To estimate the value of $K_{v}$, we assumed that the influence on the mRNA production rate $k_{n}$ (Equation 13) is primarily determined by the term $V_{n}/\left(V_{n}+K_{v}\right)$ when the cell volume increases, which we confirmed numerically (Figure S5). We calculated the fold changes of mRNA production rates ($FC_{kn}$) in cells with different volumes as

(Equation 53) $$FC_{kn}=\frac{V_{n}\left(V_{n,0}+K_{v}\right)}{V_{n,0}\left(V_{n}+K_{v}\right)}\text{,}$$

where $V_{n,0}$ is the reference nuclear volume. We fit this equation to the experimental data from^26^ to determine the value of $K_{v}$ (Figure 3B).

We performed a fitting process to determine the remaining 11 parameters using experimental data from.^14^ We searched the optimal fitting parameters starting from the biologically reasonable values inferred from the experimental data. We used the following lower indexes to indicate the values of different factors at different phases of Xrn1 depletion: “bf” (before depletion), “1”(the start of the accumulation phase), “acc” (during the accumulation phase), “2”(the start of the adaptation phase), “adp” (during the adaptation phase), “3” (the start of the reversion phase), and “rev” (during the reversion phase). Figure S10 shows a schematic of the fitting process.

We assumed that $\alpha_{y}$ is sufficiently large so that $N_{Yp}$ is virtually proportional to $k_{n}$ at any given time, i.e., $N_{Yp}\approx k_{n}/\alpha_{y}$, which ensures a constant nuclear mRNA concentration (note that if $N_{Yp}=k_{n}/\alpha_{y}$, $N_{mn}$ always equals to its steady-state value according to Equation 1).

Based on the simulations in Figure 4B, we noticed that the excess mRNA is degraded exponentially in the reversion phase since $N_{Xc}$ has reached its steady value at the beginning of the reversion phase. Therefore, we fit the data during the reversion phase (after 95 min) using the following equation:

(Equation 54) $$FC_{mt}=1+ae^{-bt}\text{,}$$

where $FC_{mt}$ represents the fold change of mRNA concentration relative to the value before perturbation at time *t*, and *a* and *b* are the parameters determined by fitting. On the other hand, since the nuclear mRNA concentration is constant throughout the process, the nuclear mRNA export rate always equals $k_{n}$. Therefore, we rewrote Equation 4 as

(Equation 55) $$\frac{dN_{mc}}{dt}=k_{n}-\beta_{m}\frac{N_{Xc}}{V_{c}}N_{mc}\text{.}$$

We then integrated Equation 55 to obtain the temporal changes in cytoplasmic mRNA:

(Equation 56) $$N_{mc_{rev}}=\frac{k_{n,rev}+Ce^{-\delta_{m,rev}t}}{\delta_{m,rev}}\text{,}$$

where $N_{mc_{rev}}$ is the cytoplasmic mRNA number during the reversion phase, $k_{n,rev}$ is the mRNA production rate during the reversion phase (which is constant in the reversion phase), and *C* is a constant. $\delta_{m,rev}$ is the mRNA degradation rate per mRNA so that $\delta_{m,rev}=\beta_{m}\frac{N_{Xc_{rev}}}{V_{c}}$, where $N_{Xc_{rev}}$ is the number of Xc during the reversion phase (which is also constant). Given that the amount of nuclear mRNA remains constant, we expressed the fold change of total mRNA as

(Equation 57) $$FC_{mt}=\frac{N_{mn}+N_{mc_{rev}}}{N_{mn}+N_{mc_{bf}}}\text{,}$$

where $N_{mn}$ is the nuclear mRNA number and $N_{mc_{bf}}$ is the cytoplasmic mRNA number before Xrn1 depletion. Using Equations 54, 55, 56, and 57, it is easy to find that $\delta_{m,rev}=b$.

Experimental data showed that $k_{n,rev}$ decreased to approximately 0.4 times the mRNA production rate before perturbation $k_{n,bf}$: $k_{n,rev}/k_{n,bf}=0.4$ (Figure 4A). Because the cytoplasmic mRNA numbers are the same between the steady-states before and after the perturbation, $k_{n,rev}/\delta_{m,rev}=k_{n,bf}/\delta_{m,bf}$. Therefore, $\delta_{m,rev}/\delta_{m,bf}=0.4$, and $N_{mc_{bf}}=k_{n,bf}/\delta_{m,bf}=0.4k_{n,bf}/\delta_{m,rev}$. Typically, the mRNA production rate of budding yeast cells ranges from about 180 to 2300 mRNA per minute.^78^ Hence, we set the mRNA production rate of WT cells before perturbation $k_{n,bf}=500$ $\min^{-1}$, from which we determined the value of $N_{mc_{bf}}$.

Next, We focused on the accumulation and adaptation phases to estimate the remaining parameters’ values. We rewrote Equation 13 as:

(Equation 58) $$k_{n}=k_{0}^{\prime }\frac{N_{Xp}}{N_{Xp}+K_{x}^{\prime }}\text{,}$$

where $K_{x}^{\prime }=K_{x}V_{n}$, and $k_{0}^{\prime }=k_{0}\frac{V_{n}}{V_{n}+K_{v}}\frac{c_{Yp}}{c_{Yp}+K_{y}}\frac{K_{m}}{c_{mn}+K_{m}}$. We assumed that $k_{0}^{\prime }$ is almost constant throughout the process so that the mRNA production rate $k_{n}$ (Equation 13) is approximately proportional to $N_{X_{p}}/\left(N_{Xp}+K_{x}^{\prime }\right)$ during the depletion experiment of Xrn1.

Calculating the analytical solutions for the time dependence of $N_{Xp}$ and $k_{n}$ was challenging, particularly for $k_{n}$ as it depends on $N_{Xp}$. To simplify the problem, we made the following approximations. Simulations in Figure 4B showed that $N_{Xc}$ increases approximately linearly in the accumulation and adaptation phases. Therefore, the transformation speed $v_{trans}=\beta_{x}N_{Xc}$ from Xc to Xp is also a linear function of time during the accumulation and adaption phase, $v_{trans}\left(t\right)=v_{trans,1}+\frac{v_{trans,3}-v_{trans,1}}{T_{acc}+T_{adp}}t$. Here, $T_{acc}$ and $T_{adp}$ are the durations of the accumulation and adaptation phases. In the steady-state before perturbation, $v_{trans,bf}=k_{n,bf}=\beta_{x}N_{Xc_{bf}}$. At the beginning of the accumulation phase, we assumed that 80% of Xc are depleted so that $N_{Xc_{1}}\approx 0.2N_{Xc_{bf}}$, therefore $v_{trans,1}\approx 0.2v_{trans,bf}$. Because the mRNA production rate and the transformation rate must balance as well in the steady state after perturbation, $v_{trans,rev}=k_{n,rev}=\beta_{x}N_{Xc_{rev}}$. According to the experimental data, $k_{n,3}=k_{n,rev}\approx 0.4k_{n,bf}$ at the end of the adaptation phase, so that $v_{trans,3}=v_{trans,rev}\approx 0.4v_{trans,bf}$. Thus, we obtained an approximate expression for the transformation speed. We also calculated the time-averaged transformation rates in the accumulation phase ($\left\langle v_{trans,acc}\right\rangle$) and the adaptation phase ($\left\langle v_{trans,adp}\right\rangle$).

Similarly, we approximated the change in $k_{n}$ in the experimental data with a combination of two linear reductions: a small slope in the accumulation phase and a large slope in the adaptation phase. Likewise, we computed the time-averaged mRNA production rates during these two phases ($\left\langle k_{n,acc}\right\rangle$ and $\left\langle k_{n,adp}\right\rangle$). With these linear approximations, we calculated the changes in $N_{Xp}$ during the accumulation and adaptation phases:

(Equation 59) $$N_{Xp_{2}}=N_{Xp_{bf}}-T_{acc}\left(\left\langle k_{n,acc}\right\rangle -\left\langle v_{trans,acc}\right\rangle \right)\text{,}$$

(Equation 60) $$N_{Xp_{3}}=N_{Xp_{2}}-T_{adp}\left(\left\langle k_{n,adp}\right\rangle -\left\langle v_{trans,adp}\right\rangle \right)\text{,}$$

where $N_{Xp_{2}}$ and $N_{Xp_{3}}$ are the numbers of Xp at the end of the accumulation and adaptation phases, respectively. Based on experimental data, the mRNA production rate at the end of the accumulation phase ($k_{n,2}$) was approximately $0.95k_{n,bf}$, while at the end of the adaptation phase, $k_{n,3}$ was around $0.4k_{n,bf}$. Using Equation 58, we wrote following equations:

(Equation 61) $$k_{n,1}=k_{0}^{\prime }\frac{N_{Xp_{bf}}}{N_{Xp_{b}f}+K_{x}^{\prime }}\text{,}$$

(Equation 62) $$k_{n,2}=k_{0}^{\prime }\frac{N_{Xp_{2}}}{N_{Xp_{2}}+K_{x}^{\prime }}\text{,}$$

(Equation 63) $$k_{n,3}=k_{0}^{\prime }\frac{N_{Xp_{3}}}{N_{Xp_{3}}+K_{x}^{\prime }}\text{.}$$

Given that $k_{n,bf}=500$ $\min^{-1}$, we solved the above equations and obtained the values of $k_{0}^{\prime }$, $K_{x}^{\prime }$, and $N_{Xp_{bf}}$.

Next, we numerically calculated the amount of mRNA accumulated during the accumulation and adaptation phases. Using $\delta_{m,bf}=\beta_{m}\frac{N_{Xc_{bf}}}{V_{c}}$, we rewrote Equation 55 as

(Equation 64) $$\frac{dN_{mc}}{dt}=k_{n}-\delta_{m,bf}\frac{N_{Xc}}{N_{Xc_{bf}}}N_{mc}\text{.}$$

Using the linear approximations of $k_{n}$ and $N_{Xc}$ in the accumulation and adaptation phase, we numerically integrated the equation to obtain the total cytoplasmic mRNA $N_{mc_{3}}$ at the end of the adaptation phase, where the total mRNA is approximately $1.3N_{mt_{bf}}$ (Figure 4A). Therefore,

(Equation 65) $$1.3=\frac{N_{mn}+N_{mc_{3}}}{N_{mn}+N_{mc_{bf}}}\text{,}$$

from which we solved for the value of $N_{mn}$.

We have so far obtained the following parameters from the experimental data of^14^: $N_{mc}$ ($N_{mc_{bf}}$), $N_{mn}$, $N_{Xp}$ ($N_{Xp_{bf}}$), $k_{0}^{\prime }$, $K_{x}^{\prime }$, and $\delta_{m}$ ($\delta_{m,bf}$) for a cell without Xrn1 depletion. We next estimated all the parameters as the starting point of the parameter search process. We set $k_{0}=6000$ $min^{-1}$, $K_{y}=100$ $fL^{-1}$, and $N_{Yp}=1\times 10^{4}$ to solve $K_{m}$ with the definition of $k_{0}^{\prime }$. We set $N_{Xn}=5000$ to solve $\alpha_{x}$ with Equation 10. We set $N_{Xc}=2\times 10^{5}$ to solve $\beta_{x}$ with Equation 11 and $\beta_{m}$ with the definition of $\delta_{m}$. We set $N_{Yp}=N_{Yn}=1000$ and solve $\alpha_{y}$ with Equation 12. We then solve $\alpha_{m}$ with Equation 8. Summing up $N_{Xn}$, $N_{Xc}$, and $N_{Xp}$, we obtained $N_{Xt}$ and similarly for $N_{Yt}$.

Finally, we optimized these 11 parameters to fit the experimental data by minimizing the weighted mean squared error (MSE) between the model predictions and the experimental data of the temporal fold changes of mRNA concentration and the mRNA production rates. This optimized set of parameters is the basic set of parameters used in our simulations (Table 1).

#### Details of numerical calculations

For the simulations of global genetic perturbations (Figures 2D, 2E, 2F, and S4), we calculated the steady-state values of mRNA concentrations, mRNA production rates, and mRNA degradation rates using Equations 8, 9, 10, 11, and 12. We randomly sampled all parameters from lognormal distributions for the blue points in Figures 2D, 2E, 2F, and S4. The CVs of parameters involved in the expression of $k_{n}$, i.e., $k_{0}$, $K_{v}$, $K_{x}$, $K_{y}$, $V_{n}$, and $V_{c}$, were set to 0.5, while the CVs of other parameters were set to 0.2. The total numbers of X and Y were also randomly sampled from lognormal distributions with a CV of 0.5. For the red points in Figures 2D, 2E, and S4, we randomly sampled $\alpha_{m}$ from a lognormal distribution with its CV equal to 1, while the CVs of other parameters in the expression of $k_{n}$, as well as the total numbers of X and Y, were set to 0.2. In these simulations, we modified $k_{0}$ to be 1/8 of the value from the set of basic parameters.

For the simulations involving changing cell volumes (Figures 3B, 3C, and 3D), we calculated the steady-state values of the mRNA number, the production rate, and the degradation rate per mRNA given a cell volume. We maintained constant concentrations of total X and Y across different volumes. To verify the constant mRNA concentration predicted by the RS model for any volume dependence of the mRNA production rate, we replace the volume-dependence term $V_{n}/\left(V_{n}+K_{v}\right)$ in Equation 13 by $10^{-5}V_{n}^{1.5}$ in Figure 3C, and $0.05\sqrt{V_{n}}\left(\sin\left(5V_{n}\right)+2\right)$ in Figure 3D. In these simulations, we modified $k_{0}$ to be 1/3 of the value from the set of basic parameters.

For the simulations involving perturbations of Pol II numbers (Figure 3E), we monitored the temporal changes in the mRNA concentration of a specific gene under different values of $K_{v}$ after silencing the expression of that gene. The parameters used in the simulations (except $K_{v}$) were optimized from the basic parameters to minimize the squared error ($E^{2}$) between the experimental and predicted degradation rates in WT cells (see details in the analysis of experimental data).

For the simulations depicting the temporal changes after acute depletion of Xrn1 (Figure 4A, 4B, 4D, 4E, and S7), we calculated the fold changes of different molecules compared to the respective homeostatic values before depletion. To mimic the acute depletion of Xrn1, we instantaneously removed 80% of Xc from the cytoplasm. In Figures 4D, 4E, and S7, two simulations are shown together in each plot. All parameters used in these simulations are the same except for the one indicated in each plot.

For the simulations examining temporal changes of mRNA with different concentrations of X or Y (Figure 5A), we monitored the changes of cytoplasmic and nuclear mRNA levels after inhibiting transcription, compared to their values before perturbation. We inhibited transcriptional by instantaneously decreasing the value of $k_{0}$ to its 50% at time 0.

For the simulations of temporal changes after complete transcription inhibition (Figure 5C), we introduced a very large $K_{v}$ to completely shut off transcription at time zero, and then calculated the temporal changes of mRNA and X.

For the simulation of the modified model with additional protein P (Figure 6B), we simulated the temporal changes of the total number of mRNA, the cytoplasmic P ($N_{Pc}+N_{Pcb}$), the nuclear P ($N_{Pn}+N_{Pnb}$), the mRNA production rate, and the mRNA degradation rate per mRNA using Equations 48, 49, 50, 51, and 52 after increasing the degradation factor $\beta_{m}$ by a factor of 10 at time 0, mimicking the globally accelerated degradation of the host mRNA during the viral infection. In these simulations, we modified $\alpha_{m}$ to be 20 times of the value from the set of basic parameters.

To simulate the extended model involving multiple distinct subsets of mRNAs (Figure 6D), we simulated the transcription of two groups of genes regulated by their own Xs and Ys. The parameters of these two RS models are set the same. We perturbed the expression of one group of genes by decreasing the number of its Xc to its 10% at time zero. We then monitored the temporal changes of the mRNA concentrations of both groups and the total mRNA concentration.
