# Supplementary material for: Homeostasis of mRNA concentrations through coupling transcription, export, and degradation
Source: iScience. 2024 Jul 18;27(8):110531. doi: 10.1016/j.isci.2024.110531 (PMC11338957; doi:10.1016/j.isci.2024.110531)
Supplement: Document S1. Figures S1–S10 and Table S1 [file mmc1.pdf]

iScience, Volume *27*

Supplemental information

Homeostasis of mRNA concentrations through coupling transcription, export, and degradation

Qirun Wang and Jie Lin

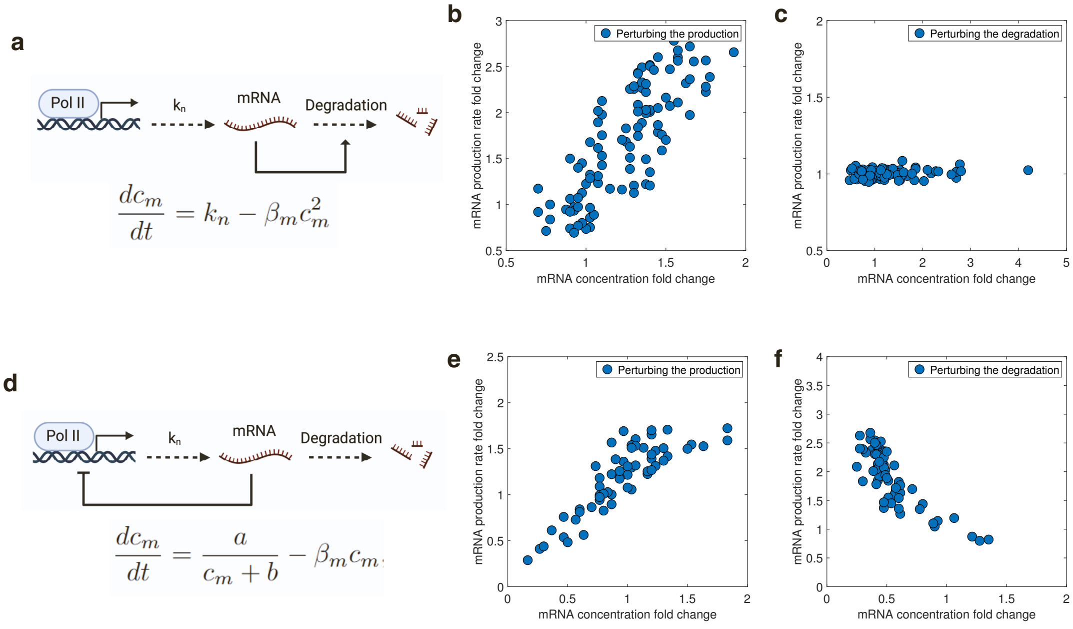

Figure S1: **Models based on mRNA feedback cannot achieve mRNA buffering, related to Figure 1.** (a) Schematic of the positive feedback model. mRNA is produced with rate k_n, and then gets degraded. The solid arrow represents the positive regulation. The equation below illustrates a specific form of positive regulation, which is used in the simulations of (b) and (c). (b) In the positive feedback model, a positive correlation exists between the mRNA production rate and mRNA concentration when the mRNA production rate k_n fluctuates. (c) In the positive feedback model, the mRNA production rate is uncorrelated with the mRNA concentration when the mRNA degradation rate $\beta_m c_m^2$ fluctuates by perturbing β_m. (d) Schematic of the negative feedback model. The solid arrow represents the negative regulation. The equation below illustrates a specific form of negative regulation, which is used in the simulations of (e) and (f). (e) In the negative feedback model, a positive correlation exists between the mRNA production rate and mRNA concentration when the mRNA production rate $k_n = a/(c_m + b)$ fluctuates by perturbing the parameter a. (f) In the negative feedback model, a negative correlation exists between the mRNA production rate and mRNA concentration when the mRNA degradation rate $\beta_m c_m^2$ fluctuates by perturbing β_m. In all scenarios, the mRNA concentration is not strictly buffered.

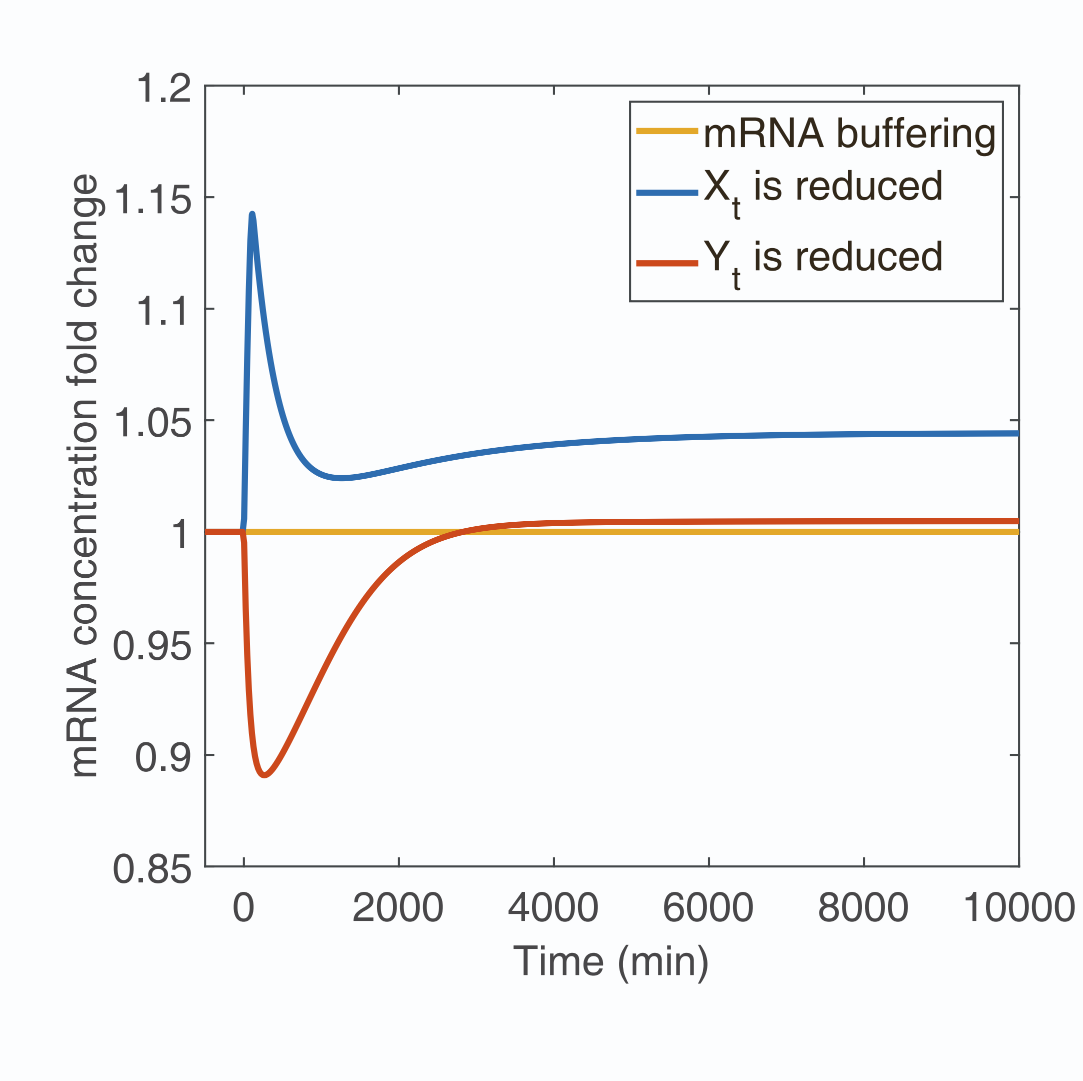

Figure S2: **Breakdown of mRNA buffering in the modified model where X is not necessary for transcription, related to Figure 1.** We simulate the modified model in which two kinds of different k_n (Eq. 16 and 17) are introduced. At time 0, we deplete either 95% Xt or 99% Yt, and monitor the temporal changes of the total mRNA levels. The fold changes represent the relative values of the perturbed cells compared to those before the perturbation.

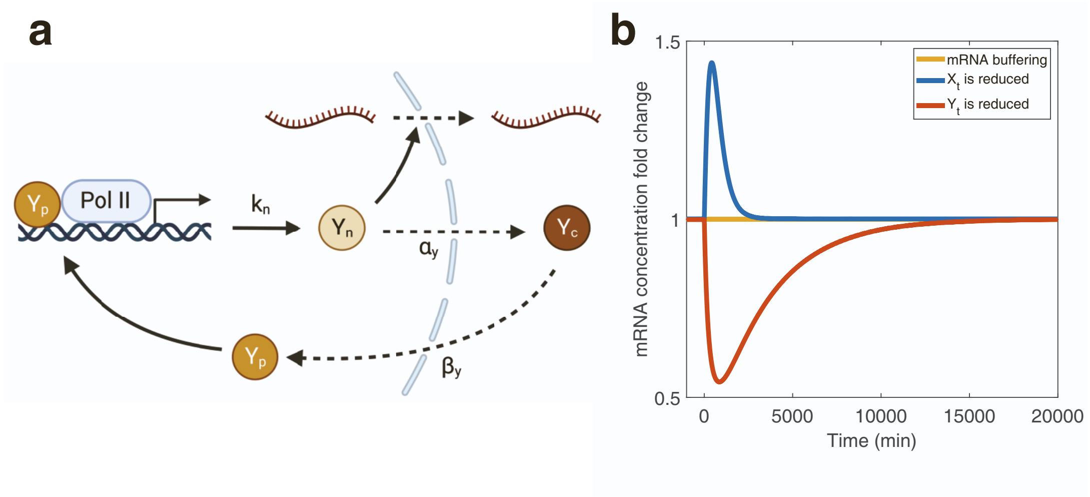

Figure S3: **mRNA buffering is still valid in the modified model where Y also shuttles, related to Figure 1.** (a) Schematic of the modified model. (b) The temporal change of mRNA concentration after the total number of X or Y is reduced. We simulated the modified model in which the dynamics of Y follows Eq. 26-28. At time 0, we depleted either 90% Xt or 90% Yt, and monitored the temporal changes of the total mRNA levels. The fold changes represent the relative values of the perturbed cells compared to those before the perturbation.

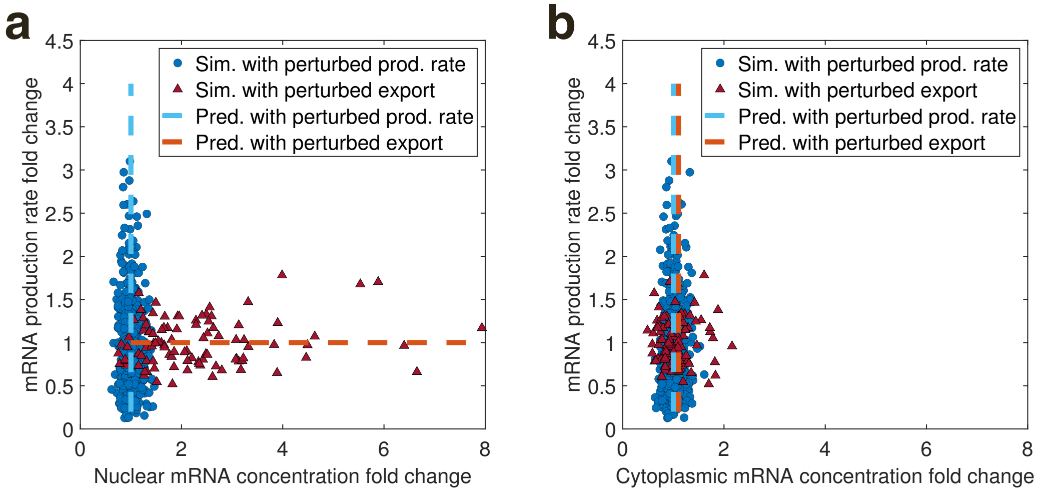

Figure S4: **Simulations of the relationships between the mRNA production rates and the mRNA concentrations without the negative feedback of nuclear mRNA on transcription, related to Figure 2.** Sim., simulation; prod., production; pred., prediction. The blue dashed line is an $x = 1$ line, and the red dashed line represents the prediction when α_m is perturbed. (a) Simulations for the nuclear mRNA concentration. The blue circles represent simulation results with multiple parameters perturbed, and the red triangles represent simulation results with α_m perturbed. The fold changes represent the relative mRNA concentrations compared to those in the control group. The same meanings also apply to (b). We randomly sampled the mRNA copy numbers from a Poisson distribution with the means equal to the predictions of the RS model, mimicking noises in gene expression. We also added Gaussian noise on top of the mRNA production rates so that the CVs equal 0.01. The same noises were also applied to (b). (b) Simulations for the cytoplasmic mRNA concentration.

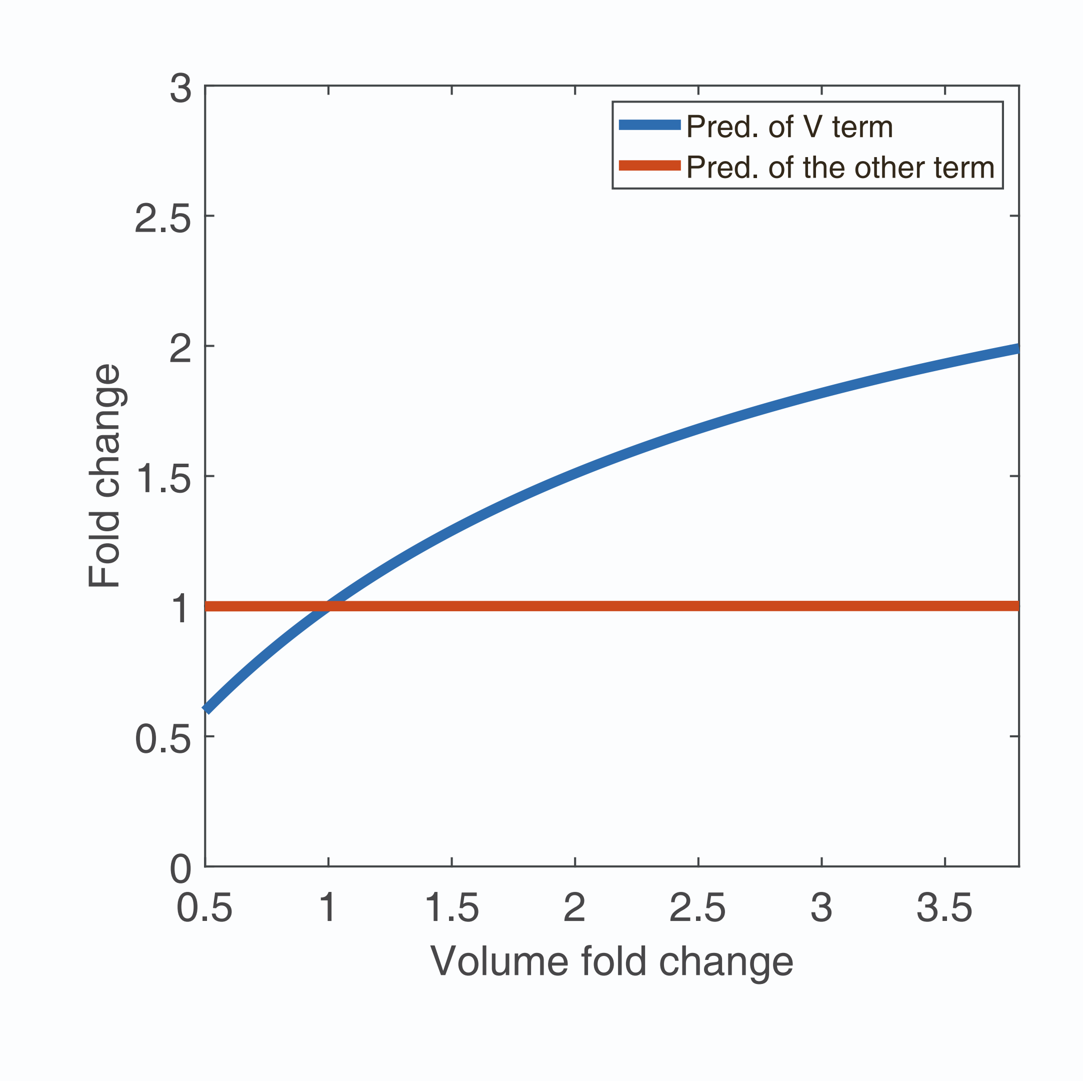

Figure S5: **The volume dependence of the two terms in k_n, related to Figure 3.** Pred., prediction. Here, the V-term is $\frac{V_n}{V_n+K_v}$, and the other term is $k_0\frac{c_{Xp}}{c_{Xp}+K_x}\frac{c_{Yp}}{c_{Yp}+K_y}\frac{K_m}{c_{mn}+K_m}$. The lines are from the same simulations as Figure 3b in the maintext. The fold changes represent the relative values of these two terms in the volume-changed cells compared to those in WT cells.

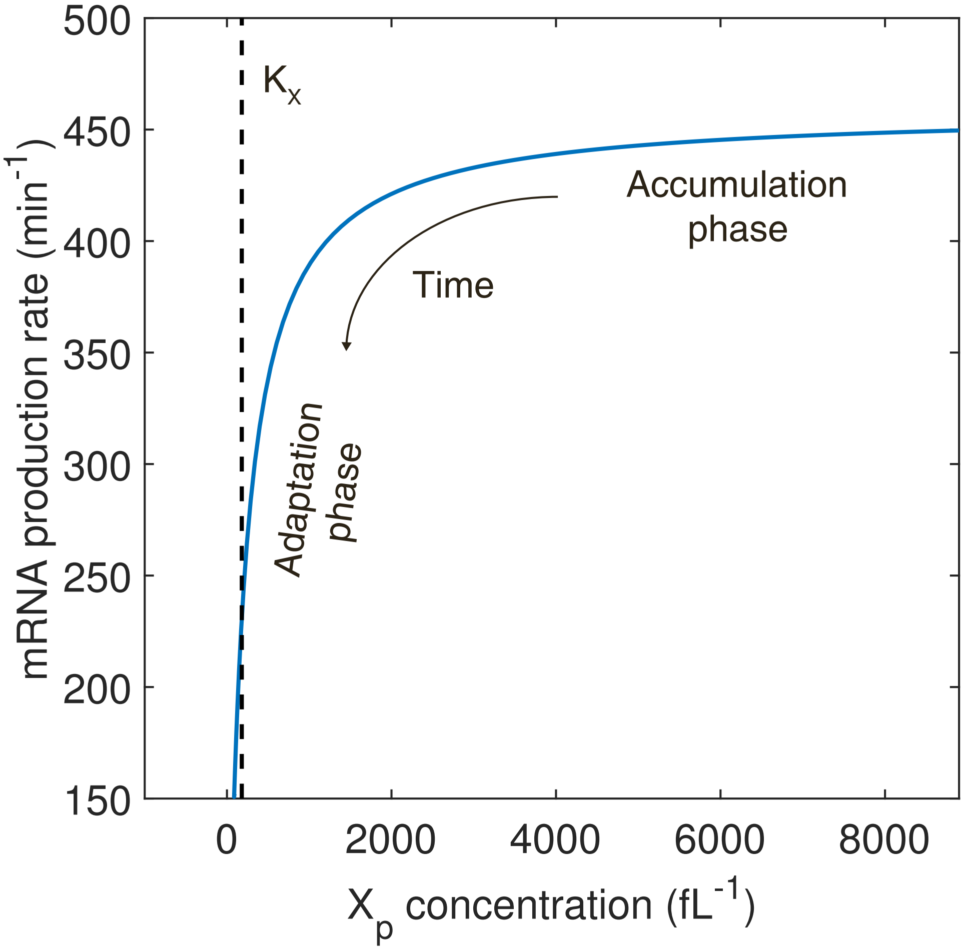

Figure S6: **Relationship between Xp and mRNA production rate in the simulation, related to Figure 4.** Xp initially decreases with a nearly constant rate until it hits the Michaelis-Menten constant K_x, which triggers a significant decrease in the mRNA production rate.

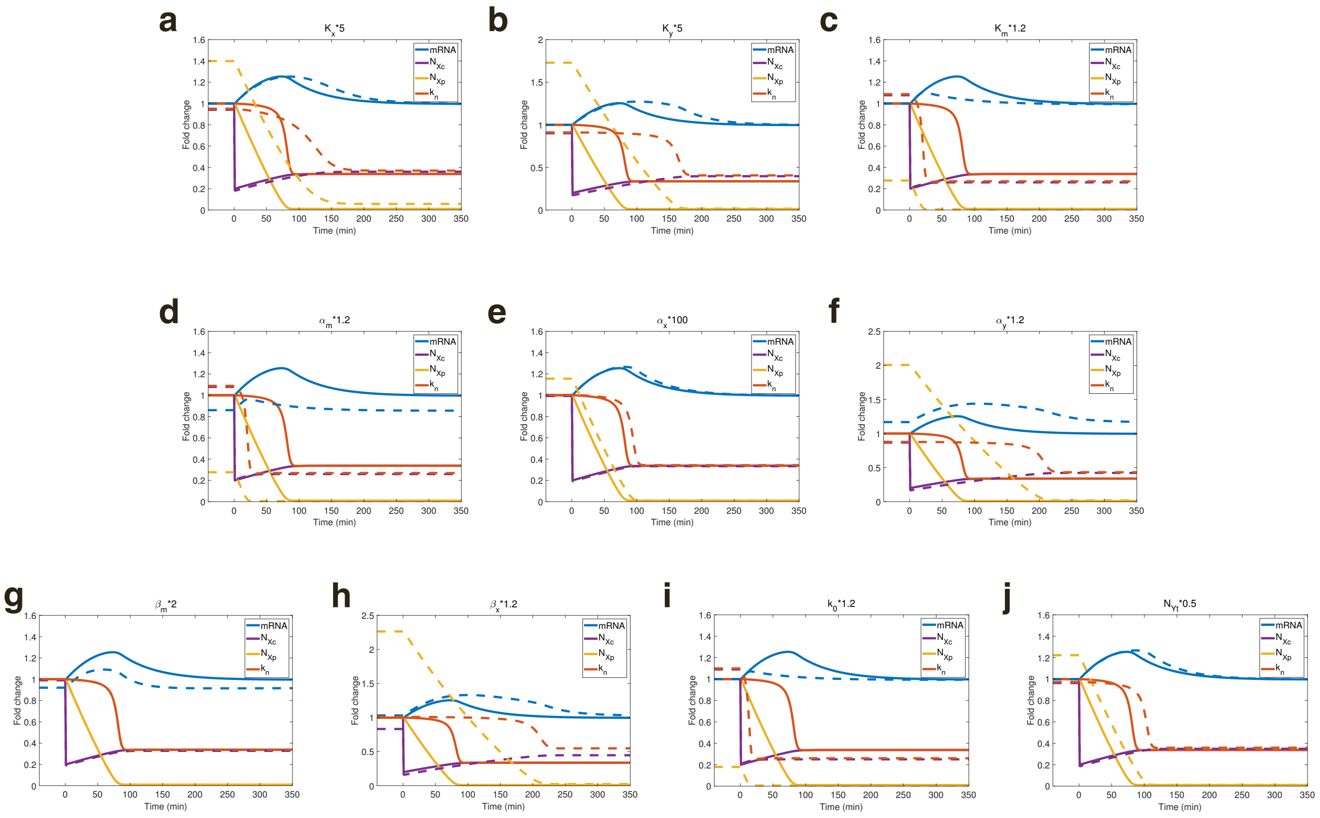

Figure S7: **Influence of different parameters on the timescales of temporal changes after depleting Xc, related to Figure 4.** The titles show how parameters are manipulated compared to those in WT cells. Solid lines show the temporal changes of WT cells, while dash lines show the temporal changes of cells in which parameters are manipulated. The fold changes represent the relative values of the WT cells and the parameter-changed cells, compared to those in the WT cells before X depletion.

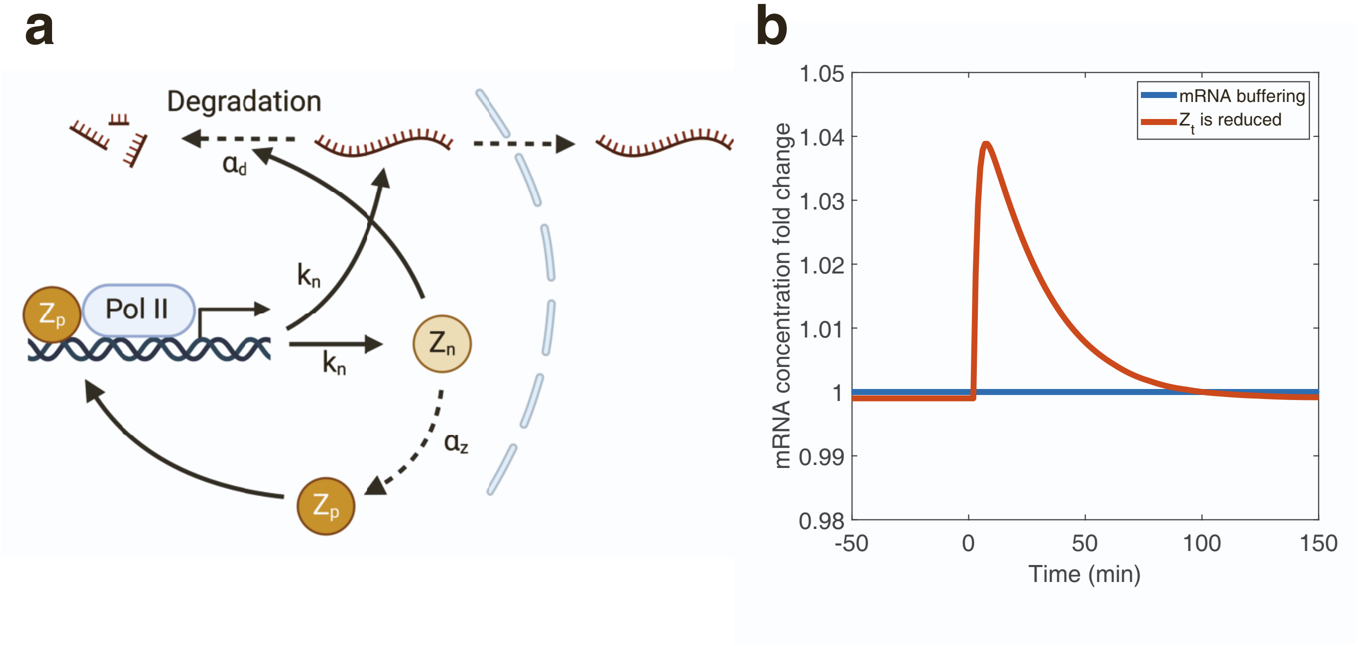

Figure S8: **mRNA buffering is still valid in the modified model, which includes protein Z that degrades nuclear mRNA, related to Figure 6.** (a) Schematic of the modified model. (b) The temporal change of mRNA concentration after the total number of protein Z is reduced. We simulated the modified model in which the dynamics of the system follows Eq. 29-30 in the main text. At time 0, we depleted 90% of Zn and Zp simultaneously and monitored the temporal changes in the total mRNA levels. The fold change represents the relative value of mRNA concentration compared to its value before depletion.

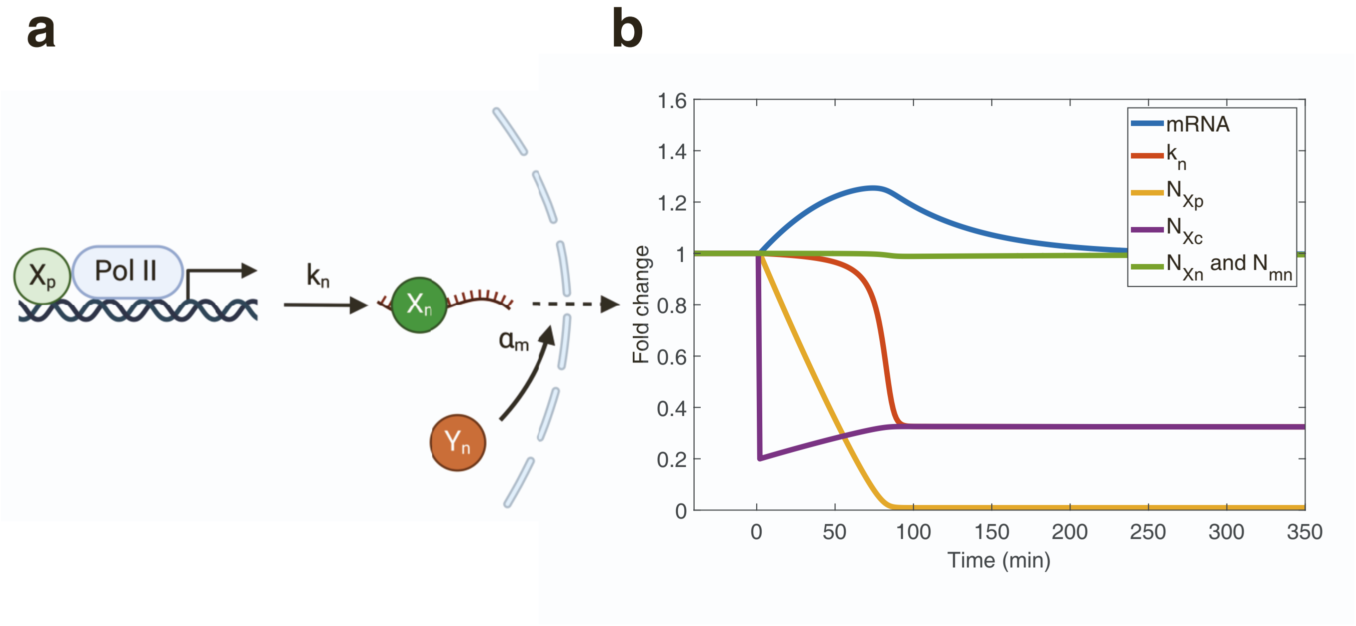

Figure S9: **mRNA buffering is still valid in the modified model where mRNA imprinting occurs, related to Figure 6.** (a) Schematic of the modified model. (b) Simulation results of the temporal dynamics of mRNA, the mRNA production rate k_n, the copy numbers of Xp, Xc, Xn (which is also the copy number of nuclear mRNA due to mRNA imprinting), and the total mRNA after acute depletion of X. The fold changes represent the values compared to the ones before depletion. The total mRNA copy number returns to the value before perturbation, with a dynamics similar to the model in the maintext (Figure 4 in the maintext).

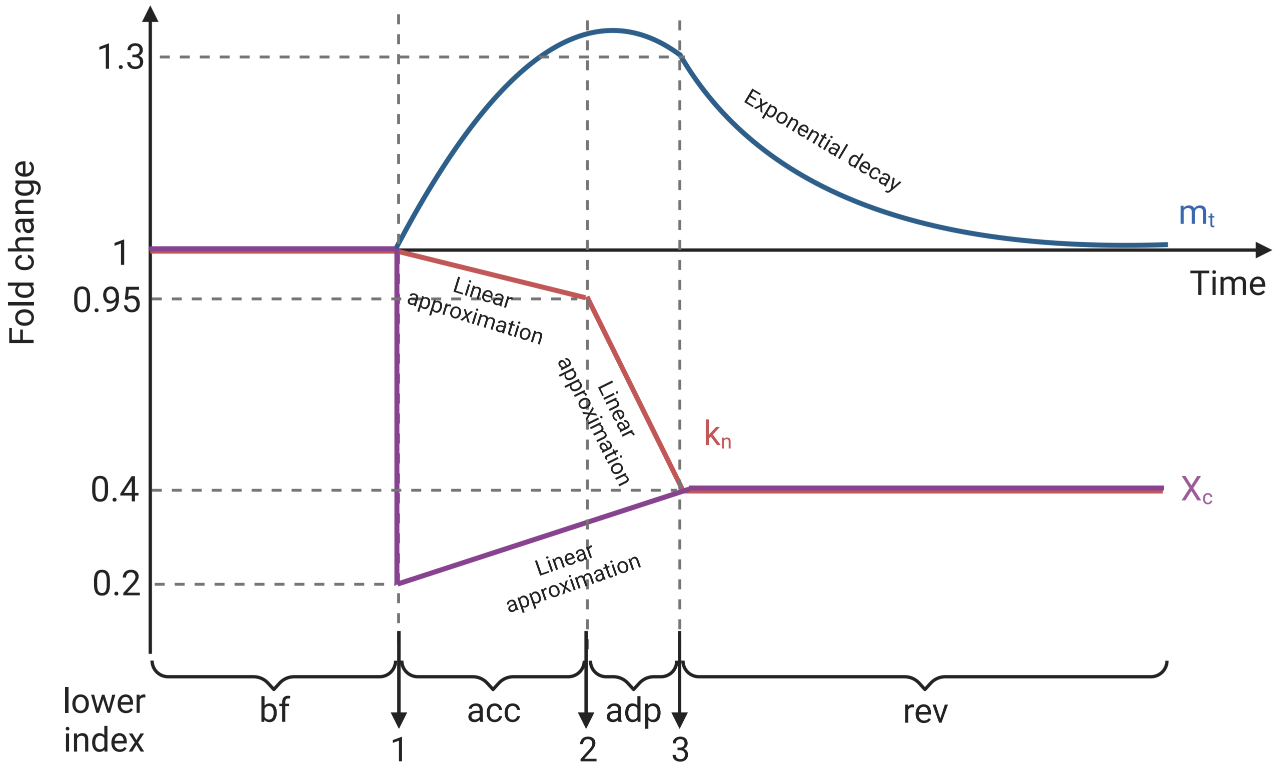

Figure S10: **Simplification of the transcription dynamics to determine fitting parameters, related to STAR Methods.** We used an exponential decay model to fit parameters during the reversion phase. We used linear approximations to model the dynamics of Xc and k_n during the accumulation and adaptation phases. The lower indexes used in Methods at different times are shown at the bottom of the schematic.

Table S1: Candidates of X and Y in *S. cerevisiae*, related to Figure 5.

Gene name	Functions as	Gene name	Functions as	Gene name	Functions as	Gene name	Functions as
CAF40	X	MOT2	X	NUP84	Y	SPN1	Y
CCR4	X	NAB2	X and Y	NUP85	Y	SPT6	Y
CDC36	X	NAP1	Y	PHO85	Y	SSL2	Y
CDC39	X	NOT3	X	POP2	X	SSN3	X
CLA4	Y	NOT5	X	RAT1	X	SUS1	X and Y
DCP2	X	NUP100	Y	RMD9	X	THO2	Y
HMT1	Y	NUP120	Y	RPB4	X and Y	UTP8	Y
HPR1	Y	NUP133	Y	RPB7	X	UTP9	Y
HTZ1	X	NUP145	Y	RSP5	Y	WTM1	Y
ISW1	Y	NUP159	Y	RTG2	Y	XRN1	X
MGA2	X	NUP42	Y	SEC13	Y	YAR1	Y
MLP2	Y	NUP53	Y	SGF73	Y	YGR122W	X
